# Can a COVID-19 vaccination program guarantee the return to a pre-pandemic lifestyle?

**DOI:** 10.21203/rs.3.rs-200069/v1

**Published:** 2021-02-09

**Authors:** Juan Yang, Valentina Marziano, Xiaowei Deng, Giorgio Guzzetta, Juanjuan Zhang, Filippo Trentini, Jun Cai, Piero Poletti, Wen Zheng, Wei Wang, Qianhui Wu, Zeyao Zhao, Kaige Dong, Guangjie Zhong, Cécile Viboud, Stefano Merler, Marco Ajelli, Hongjie Yu

**Affiliations:** 1. School of Public Health, Fudan University, Key Laboratory of Public Health Safety, Ministry of Education, Shanghai, China; 2. Bruno Kessler Foundation, Trento, Italy; 3. Division of International Epidemiology and Population Studies, Fogarty International Center, National Institutes of Health, Bethesda, MD, USA; 4. Department of Epidemiology and Biostatistics, Indiana University School of Public Health, Bloomington, IN, USA; 5. Laboratory for the Modeling of Biological and Socio-technical Systems, Northeastern University, Boston, MA, USA; 6. Shanghai Institute of Infectious Disease and Biosecurity, Fudan University, Shanghai, China; 7. Department of infectious diseases, Huashan Hospital, Fudan University Shanghai, China

## Abstract

COVID-19 vaccination programs have been initiated in several countries to control SARS-CoV-2 transmission and return to a pre-pandemic lifestyle. However, understanding when non-pharmaceutical interventions (NPIs) can be lifted as vaccination builds up and how to update priority groups for vaccination in real-time remain key questions for policy makers. To address these questions, we built a data-driven model of SARS-CoV-2 transmission for China. We estimated that, to prevent local outbreaks to escalate to major widespread epidemics, stringent NPIs need to remain in place at least one year after the start of vaccination. Should NPIs be capable to keep the reproduction number (R_t_) around 1.3, a vaccination program could reduce up to 99% of COVID-19 burden and bring R_t_ below the epidemic threshold in about 9 months. Maintaining strict NPIs throughout 2021 is of paramount importance to reduce COVID-19 burden while vaccines are distributed to the population, especially in large populations with little natural immunity.

## Reporting summary

Further information on research design is available in the Nature Research Reporting Summary linked to this paper.

The novel coronavirus disease 2019 (COVID-19) pandemic is far from over with cases still surging in many countries across the globe^1^. In 2020, epidemic suppression and/or mitigation have relied on non-pharmaceutical interventions (NPIs), including social distancing, school closure, masking, and case isolation. Although effective and widely adopted to limit SARS-CoV-2 transmission and reduce COVID-19 burden, these interventions entail enormous economic costs and negatively affect quality of life^2^. Additionally, in many countries, relaxation of NPIs has led to a resurgence of the epidemic as no location has reached herd immunity thus far^3^ – even in Manaus, Brazil where it is estimated that over >70% of the population has been naturally infected, the epidemic is seemingly not over^4^.

Effective vaccines against COVID-19 remain the only foreseeable means of both containing the infection and returning to pre-pandemic social and economic activity patterns. Globally, several vaccines have been licensed, and vaccination programs have been initiated in several countries including China^5^. However, in the near future, the projected global production and delivery capacities are likely to be inadequate to provide COVID-19 vaccines to all individuals who are still susceptible to SARS-CoV-2 infection^3^. The effectiveness of COVID-19 vaccination campaigns will depend on several factors, including vaccine supply, willingness to receive the vaccine, and strategies for vaccine allocation and deployment^6^. In particular, estimating whether and when NPIs can be lifted while vaccination campaigns are ongoing is a top priority for policy making. Moreover, optimal strategies for vaccine allocation in a shifting landscape of infections are urgently needed as well.

In this study, we aim to address these questions by using China as a case study. To do so, we build an age-structured stochastic model to simulate SARS-CoV-2 transmission in mainland China, based on a susceptible-infectious-removed (SIR) scheme (Extended Data Fig. 1). We account for heterogeneous mixing patterns by age^7^ and progressive vaccine deployment among different population segments based on a broadly accepted priority scheme (essential workers, older adults and individuals with underlying conditions, etc.). Further, we overlay a disease burden model on the transmission model to estimate the number of symptomatic cases, hospitalizations, ICU admissions, and deaths under different vaccination scenarios and based on empirical data^8–13^. The resulting model is informed by data on COVID-19 natural history, age-mixing patterns specific to China in the pandemic period, and the size of the different vaccination targets in the Chinese population (e.g., individuals with pre-existing conditions). We also leverage data on the Chinese healthcare system to estimate vaccine administration capacity. A summary of model parameters and data sources is presented in Extended Data Table 1. Model details are described in Supplementary Information File 1.

We considered a baseline reactive vaccination scenario where: 1) vaccination starts 15 days after an outbreak triggered by 40 breakthrough imported COVID-19 infections; 2) vaccine efficacy (VE) against SARS-CoV-2 infections for a two-dose schedule is set at 80%; 3) vaccination coverage is capped at 70%; 4) 6 million doses are administered daily (4 per 1,000 individuals, informed by 2009 influenza pandemic vaccination in China^14^, the ongoing COVID-19 vaccination program in Beijing^15^, estimates of vaccine supply till 2021 in China^16^); 5) the first priority target consists of older adults and individuals with underlying conditions (descriptions in details shown in Supplementary Information File 1); 6) there is no prior population immunity from natural infection, which aligns with the situation in most of China where there has been little circulation of SARS-CoV-2 in 2020^3^; 7) we assume an initial effective reproductive number R_t_ =2.5 homogeneous across age groups at the start of the outbreak, in the absence of NPI and vaccination; and 8) we let the model run for two years.

**Figure 1.**
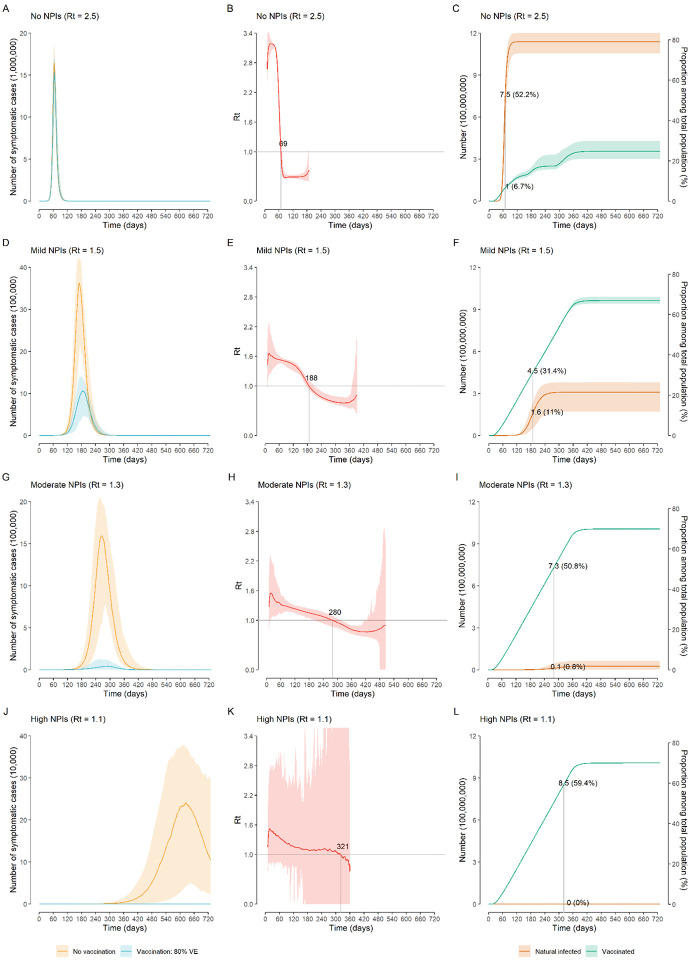
Time series of symptomatic cases, effective reproductive number R_t_, and population infected and vaccinated. A) Number of symptomatic cases over time as estimated in the no-NPIs scenario (initial R_t_=2.5) in the absence/presence of vaccination; B) Net reproduction number R_t_ over time, as estimated from symptomatic cases in the no-NPIs scenario in the presence of vaccination; C) Absolute numbers and proportion of the Chinese population infected and vaccinated over time in the no-NPIs scenario in the presence of vaccination; D)-F): as A-C but for the mild NPIs scenario (initial R_t_=1.5); G)-I): as A-C but for the moderate NPIs scenario (initial R_t_=1.3); J)-L): As A-C but for the high NPIs scenario (initial R_t_=1.1). Line denotes median, and shadow denotes quantiles 0.025 and 0.975.

**Figure 2.**
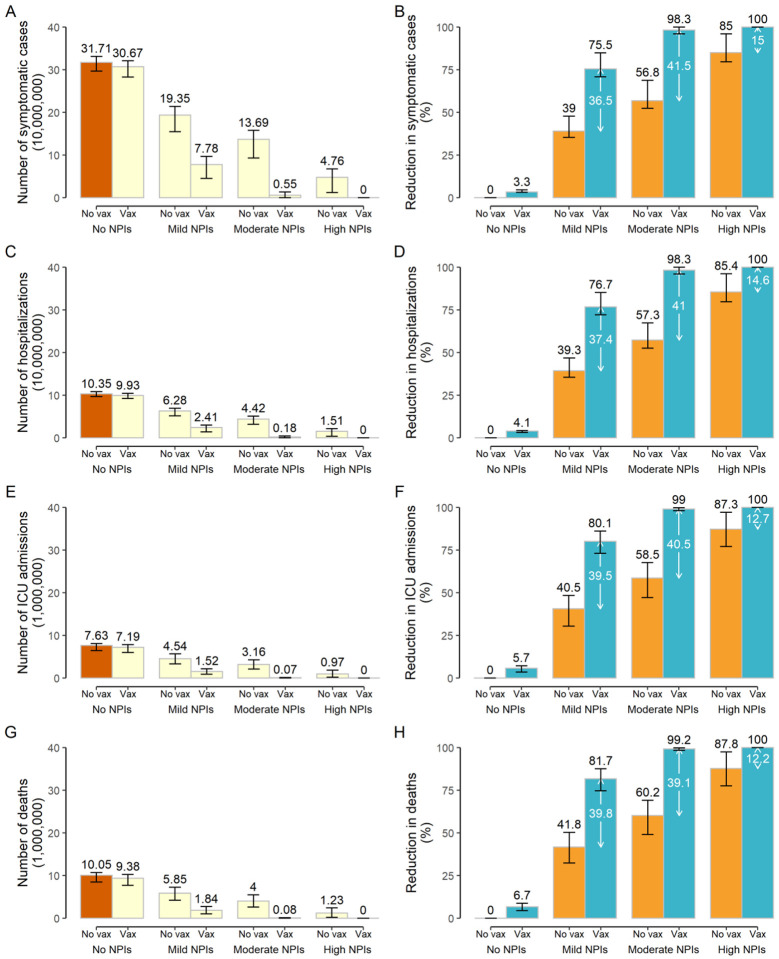
Burden of COVID-19 in the main analysis. A) Cumulative number of symptomatic cases as estimated under the different scenarios in the absence/presence of vaccination over the simulated 2-year period. No vaccination + no NPIs with R_t_=2.5 at the beginning of transmission is called *reference scenario*, described using dark brown bars. Light yellow bars indicate scenarios including vaccination and/or different levels of NPIs. B) Reduction in the cumulative number of symptomatic cases with respect to the *reference scenario*. Orange bars and black values indicate the contribution of NPIs, blue bars and black values indicate the overall contribution of vaccination and NPIs, while the white values indicate net contribution of vaccination; C)-D) As A-B but for hospitalized cases; E)-F) As A-B but for cases admitted to ICU; G)-H) As A-B but for deaths. Number denotes median, and error bars denote quantiles 0.025 and 0.975.

In the absence of NPIs, the vaccination program is too slow to lower and delay the epidemic (Fig. 1A) and does not effectively reduce COVID-19 burden. R_t_ falls below the epidemic threshold (<1) 69 days after the epidemic start (Fig. 1B), but this is primarily attributable to immunity gained through natural infection rather than vaccination. Indeed, in this time frame, 52.2% of population gets infected, while only 6.7% of population has been vaccinated (Fig. 1C). The cumulative disease burden of COVID-19 over a 2-year period only decreases by 3.3%−6.7% compared to a reference scenario where there is no vaccination and no NPIs, leading to 306.73 million (95%CI, 282.68–320.60) symptomatic cases, 99.25 million (92.55–104.51) hospitalizations, 7.19 million (6.00–7.83) ICU admissions, and 9.38 million (7.70–10.26) deaths (Fig. 2).

Provided that NPIs are in place and can keep R_t_ at 1.3 in the absence of vaccination (*moderate NPIs scenario*), initiating a vaccination program could reduce the COVID-19 burden by about 99% compared to the *reference no-vaccination scenario*, with 5.46 million (2.47–13.36) symptomatic cases, 1.77 million (0.83–4.40) hospitalizations, 73,500 (7,300–152,100) ICU admissions, and 76,700 (8,200–165,700) deaths (Fig. 2). In this context, vaccination decreases the COVID-19 burden by about 40% (Fig. 2) compared to a situation with moderate NPI alone, and R_t_ falls below the epidemic threshold about 9 months after the epidemic start (Fig. 1). At the time that R_t_ falls below 1, we estimate that 50.8% of the total population would have been vaccinated, while 0.8% would have been naturally infected (Fig. 1G–I). This highlights that, although in the long-term vaccination can ultimately lead to the suppression of COVID-19, it is necessary to maintain the NPIs currently in place for one year after the onset of vaccination. For instance, if NPIs are relaxed 9 months into the vaccination program, allowing a 25% increase in SARS-CoV-2 transmissibility, the cumulative death toll could increase by three folds from 76,700 to 318,300. In contrast, there is a small increase in cumulative deaths to 93,500 if NPIs are relaxed one year after vaccination (Extended Data Fig.2–3). Earlier or more drastic relaxations of NPIs lead to substantial increases in deaths (Extended Data Fig.2–3).

A combination of more stringent NPIs (i.e., capable of keeping R_t_ =1.1) and vaccination (*vax + high NPIs* scenario) could suppress the epidemic, with <2,300 symptomatic cases, and <50 deaths on average. Although the majority of the reduction of COVID-19 burden is ascribable to NPIs in this case (over 85%), the deaths averted due to vaccination are about 1.2 million (Fig. 1J–L, and Fig. 2).

If we consider a set of mild NPIs (*vax + mild NPIs* scenario), even a relatively low initial reproduction number under NPIs of R_t_=1.5 could still lead to a disastrous epidemic, with nearly two million deaths. Despite the high death toll of the resulting epidemic, NPIs and vaccination would jointly reduce around 80% of the disease burden compared to a non-NPI non-vaccination scenario (namely, 239 million symptomatic cases and 8.2 million deaths averted) (Fig. 1D–F, and Fig. 2).

## Impact of vaccine distribution capacity

**Figure 3.**
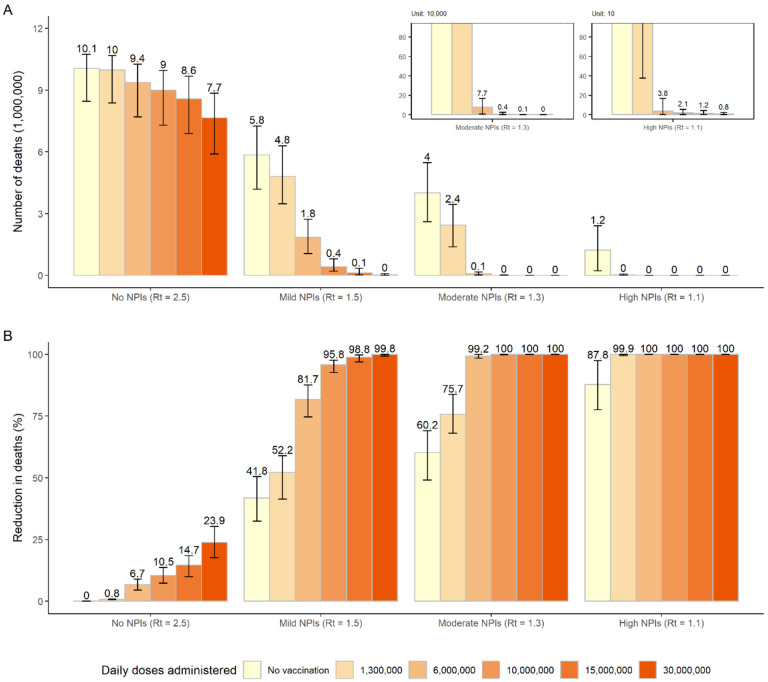
Impact of daily vaccine administration capacity on COVID-19 deaths. A) Cumulative number of COVID-19 deaths (millions) as estimated in the different scenarios under progressively increasing values of the daily vaccination capacity; B) Proportion of deaths averted compared to the *reference scenario*, i.e., *no vaccination + no NPIs* with R_t_=2.5 at the beginning of transmission. Number denotes median, and error bars denote quantiles 0.025 and 0.975.

Should the daily vaccination rollout be limited to 1.3 million doses (1 per 1,000 individuals, a slower rate than during the 2009 pandemic), vaccination would not effectively reduce COVID-19 related deaths unless there was adoption of stringent NPIs. In an optimistic scenario where vaccination capacity reaches 10 million doses administered per day (7 per 1,000 individuals), vaccination would reduce COVID-19 related deaths to <5,000 for moderate NPIs and <30 for high NPIs. Should the daily vaccination capacity be increased to 15 million doses (10 per 1,000 individuals), vaccination could effectively reduce deaths to <100,000 (similar to the annual influenza-related death toll in China^17^) even in the presence of mild NPIs. However, even if the daily vaccination capacity could be increased to 30 million doses (20 per 1,000 individuals), in the absence of NPIs, we estimate that over 7.7 million deaths would still occur (Fig. 3). Similar patterns are estimated for the number of symptomatic cases, hospitalizations and ICU admissions (Extended Data Fig.4–6).

Increasing daily vaccination capacity could largely shorten the time needed to control SARS-CoV-2 transmission. For instance, when considering a daily capacity of 10 million and 15 million doses and moderate NPIs, R_t_ would drop below 1 about 8 and 6 months respectively after epidemic onset (to be compared to the 9.3 months estimated with the baseline capacity of 6 million doses). At that time, over 60% of the population would be vaccinated and ≤0.1% would be naturally infected.

As highlighted in vaccination studies in the UK and Australia^18–20^, in the race between the vaccination campaign to build population herd-immunity and the progress of the epidemic, the speed of vaccine deployment is critical. In the routine National Immunization Program, an average of 1.4 million doses are administered in China per day^21,22^, while during the 2009 influenza pandemic a maximum of 3 million daily doses were administered^14^. Considering that the willingness to be vaccinated against COVID-19 is higher than that for the 2009 influenza pandemic^23^, and that the vaccine distribution capacity is likely to be improved as well (e.g., 3–5 folds increase in current COVID-19 vaccination campaign in Beijing^15^), we consider the capacity of COVID-19 vaccination services could be scaled up to 6 million doses administered per day in the baseline analysis. Several manufacturers state that a total of 2.1 billion doses of COVID-19 vaccine could be produced in 2021, equivalent to about 6 million doses per day, which could be enough to cover 75% of the Chinese population^16^. Even if these candidate vaccines could be licensed and manufactured smoothly, it would take about one year to vaccinate 70% of the general population.

In addition, limited vaccine production capacity, particularly at the initial stage, could slow the speed of vaccine rollout. Chinese media reported that the government planned to administer 100 million doses for emergency use by February 15, 2021^24^, with an average of less than 2 million doses per day. Slower rates of vaccine production and administration may result in a longer period of COVID-19 transmission. It is thus crucial to keep monitoring local outbreaks and invest resources in outbreak management in order to keep R_t_ close to the epidemic threshold (or, at most, not to exceed 1.3) at least for the next 1–2 year. Moreover, the development of detailed logistical plans and tools to support an increased vaccination capacity as well as effective logistic (vaccine transport, storage, and continuous cold-chain monitoring) are key factors for a successful mass vaccination campaign.

## Vaccination prioritization

**Figure 4.**
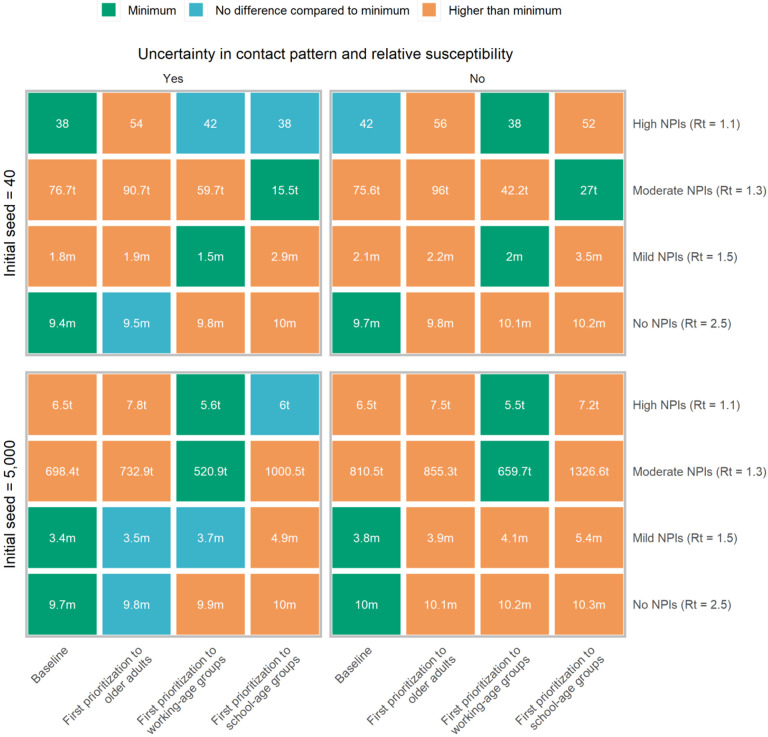
Best prioritization strategy to achieve the minimal COVID-19 deaths. Initial cases denote breakthrough COVID-19 cases, which initiates the epidemic. We consider the impact of uncertainty in contact patterns and relative susceptibility on prioritization, and use their mean values as well. Baseline denotes first prioritizing older adults and individuals with underlying conditions. Number in the box denotes the death toll (median), with t representing thousand and m representing million. Minimum denotes the lowest deaths in each scenario on the basis of median value. We compare other strategies to that with minimum deaths using rank sum test. E.g., in the context of initial cases=5,000, R_t_=1.5 and using mean values of contact patterns and relative susceptibility, the baseline is the optimal strategy to minimize deaths.

We consider alternative vaccination scenarios that prioritize essential workers (staff in the healthcare, law enforcement, security, community services, and individuals employed in cold chain, etc.) to maintain essential services and then explore different prioritization strategies for the rest of the population. Our results suggest that the relative timing of the epidemic and of the vaccination rollout play a key role in determining the most effective strategy. In particular if we consider vaccination to start at about the same time as an outbreak (i.e., two weeks after 40 cases are detected – as in the other analyses presented in the main text), there is no clear prioritization strategy that minimize deaths, as the outcome of the vaccination campaign heavily depends on the timing at which the epidemic unfolds (Fig. 4 and Extended Data Fig. S7–8). Instead, if the epidemic is already underway when the vaccination campaign starts (>5,000 cases), prioritizing working-age groups minimizes the number of deaths when R_t_≤1.3. In contrast, prioritizing older adults and individuals with underlying conditions is more effective when R_t_≥1.5 (direct benefits are higher, Fig. 4 and Extended Data Fig. S7–8). Two results are independent of the adopted prioritization strategy: i) if R_t_≥1.5, then an epidemic cannot be avoided; and ii) when R_t_ =1.1, over 99% of deaths can be averted (Extended Data Fig. S7–8).

Bubar K, et al. evaluated COVID-19 vaccine prioritization strategies and found that prioritizing older adults is a robust strategy to minimize deaths across countries when R_t_=1.5, while prioritization shifted to 20–49 years group when R_t_=1.15^25^. The broad scope of this multi-country analysis does not account for features of COVID-19 epidemiology and vaccination program that are unique to China. In particular, differently from most countries where SARS-CoV-2 is widespread, several rounds of lockdowns have already been required, and natural immunity is building up, China has been able to suppress SARS-CoV-2 transmission for most of 2020. As a result, prior immunity is very low, thus calling for specifically tailored analysis.

Our finding confirms that if NPIs can maintain transmission rates at low levels during the vaccination campaign, strategies that target indirect benefits do better. If transmission rates remain high, strategies maximizing direct benefits will perform best. Given that China is doing so well in clamping down transmission by enforcing strict NPIs, vaccinating working age adults may generally be a better option. In most other countries, however, vaccinating older adults would be expected to save more lives^25^.

## Alternative vaccination parameters and scenarios

To evaluate the impact of baseline assumptions on our results, we conduct comprehensive sensitivity analyses (SE) for R_t_ fixed to 1.3 (moderate NPIs). Provided that vaccination can only protect against illness (*SE18*) but not SARS-CoV-2 infections, COVID-19 related deaths increase by 33 folds with respect to the baseline: from 76,700 to 2.66 million. In this case, maintaining stringent NPIs measures in place for a prolonged time horizon would be necessary as such vaccine would not be effective to suppress transmission (as reported in previous studies^26^). Assuming a shorter duration of vaccine-induced protection of 6 months (*SE16*) instead of a lifelong protection (i.e., longer than the 2-year time horizon considered, Extended Data Fig. S9) has a similarly large effect on projections (S13).

Other factors such as vaccine coverage (*SE8* and *SE9*), excluding detected symptomatic cases from vaccination (*SE10* and *SE11*), the time interval between two doses (*SE12*), and assuming an all-or-nothing vaccine (*SE19*), do not substantially affect estimates of deaths (Extended Data Fig. S9) and symptomatic infections (Extended Data Fig. S10). A similar trend is observed for hospitalized cases and ICU admissions.

Using a stochastic dynamic model of SARS-CoV-2 transmission in combination with a COVID-19 burden model, we estimate the impact of a COVID-19 vaccination program in the absence or presence of NPIs on SARS-CoV-2 infections, symptomatic cases, hospitalizations, ICU admissions, and deaths in China. We find than in the absence of NPIs, and independently of the vaccine prioritization strategy and capacity of the vaccination campaign, timely rollout of an effective vaccine (VE =80%) would not be enough to prevent a local outbreak to escalate to a major widespread epidemic. Provided that NPIs are in place and capable to bring R_t_ to 1.3, a daily vaccine rollout of 4 doses per 1,000 individuals could reduce around 99% of COVID-19 burden, and bring R_t_ below the epidemic threshold about 9 months after the start of the vaccination campaign. A relaxation of NPIs that bring the value of R_t_ to 1.5 could not prevent sustained epidemic growth which would cause 1.8 million deaths. A net reproduction number of 1.5 could only be sustained when accompanied by an improvement of the vaccine administration capacity up to 10 doses per 1,000 individuals per day. Relaxation of NPIs in the first 6–9 months of vaccine roll out could lead to substantial increases of COVID-19 burden.

Our study proposes a general framework to evaluate the impact of COVID-19 vaccination programs in the absence/presence of NPIs and to explore priority target populations to minimize multiple disease outcomes. The proposed modeling framework is easily adaptable to other country-specific contexts, including the susceptibility of the local population^3^, local risk of transmission and implemented NPIs^27^, efficacy of different vaccines^28–31^, vaccine supply and capacity of immunization services^6^, and the objectives of the pandemic responses.

Our study has a number of limitations. First, we integrated the impact of NPIs through a simple reduction in the value of R_t_ at the beginning of the outbreak, homogeneously across age groups. However, our analysis does not suggest which combination of NPIs should be adopted to lower R_t_ to a certain level, and how this would affect transmission rates in different age groups. Li, et al, estimated that individual NPIs, including school closure, workplace closure, and public events bans, were associated with reductions in R_t_ of 13–24% on day 28 after their introduction^32^. Further studies are needed to pinpoint the specific NPIs to be adopted in parallel with the vaccination campaign. Second, in China, vaccines have not been licensed for older adults and children, so we assume a 50% lower or equivalent VE for them compared to other adults. Although we show that variations in these rates do not substantially affect the overall effect of the vaccination campaign, further data on age-specific vaccine efficacy could help refine priority groups. Third, we assumed that immunity after natural infections lasts more than the time horizon considered (two years). If this is not the case, waning of immunity would inflate the rate of susceptible individuals and thus require booster vaccinations. This could become an issue with the emergence of immune-escape variants, as reported in South Africa^33^. Given limited information at this stage, we did not consider this scenario in our analyses, but this is an important area of future research.

Vaccination alone could substantially reduce COVID-19 burden, but in the foreseeable future may not be enough to prevent local outbreaks to escalate to major widespread epidemics due to limitation in the vaccine production and supply (particularly at the initial stage of the vaccination), as well as the capacity of vaccination system. This is especially relevant in contexts where most of the population is still susceptible to SARS-CoV-2 infection, as it is the case in most of China. Maintaining NPIs such as social distancing, case isolation, and careful contact tracing, wearing masks, increased teleworking and limitation on large gatherings, is necessary to prevent the resurgence of COVID-19 epidemics until a sufficiently high vaccine coverage is reached.

## Methods

### SARS-CoV-2 transmission and vaccination models

We developed a model of SARS-CoV-2 transmission and vaccination, based on an age-structured stochastic susceptible-infectious-removed (SIR) scheme, accounting for heterogeneous mixing patterns by age as estimated in Shanghai^7^. The Chinese population was distributed in 18 age groups (17 5-year age groups from 0 to 84 years and one age group for individuals aged 85 years or older)^34^. Each age group was further split into two subgroups: individuals with or without underlying conditions, where the former were considered to be associated with an increased risk of severe outcome of COVID-19^35^.

In the main analysis, susceptibility to SARS-CoV-2 infection was assumed to be heterogeneous across ages. Children under 15 years of age were considered less susceptible to infection compared to adults aged 15 to 65 years, while the elderly more susceptible^36,37^. Homogeneous susceptibility across age groups was explored in sensitivity analysis (***SE1***). Asymptomatic and symptomatic individuals were assumed to be equally infectious^36,37^, and infectiousness was also assumed to be the same across age groups^36,37^.

Vaccine is administered with a two-dose schedule. In the baseline model, we assumed that: i) vaccination reduces susceptibility to SARS-CoV-2 infection; ii) only susceptible individuals are eligible for vaccination, i.e., we excluded all individuals that have experienced SARS-CoV-2 infection; iii) duration of vaccine-induced protection lasts longer than the time horizon considered (2 years).

The baseline model is schematically represented in Extended Data Fig. 1 and it is described by differential systems presented in Supplementary File 1–2.

### Model initialization

In China, the first pandemic wave of COVID-19 was controlled by intense NPIs^38,39^. Almost the entire population of mainland China is still susceptible to COVID-19. As such, the model is initialized with a fully susceptible population (23).

China has been facing mounting pressure of imported COVID-19 cases. Containment of COVID-19 has been possible only through a combination of measures such as complete- or partial-lockdown, citywide mass-screening using reverse-transcriptase–polymerase-chain-reaction (RT-PCR) testing, tracing of contacts and contacts of contacts of COVID-19 cases, which were promptly applied wherever COVID-19 transmission has popped up in mainland China^40^. Despite all the efforts, containment of COVID-19 appears a whack-a-mole game and sporadic outbreaks inevitably occur. Simulations are thus initialized with 40 cases, roughly corresponding to the number of cases with symptoms onset in Beijing before the detection of a local outbreak in June 11, 2020.^41^

### Vaccination scenarios

To explore the impact of vaccination, we ran a set of simulations in which neither NPIs nor vaccination are implemented as a *reference scenario* (*no vax + no NPIs*, i.e., effective reproductive number R_t_=2.5 at the beginning of simulations^11,38,42^), and compared it with a scenario in which vaccination only is implemented (*vax + no NPIs*). Further, we considered different sets of simulations in which NPIs are used to bring R_t_ respectively down to 1.5 (mild NPIs), 1.3 (moderate NPIs), and 1.1 (high NPIs), with (*vax + mild/moderate/high NPIs*) or without vaccination program (*no vax* + *mild/moderate/high NPIs*). In the main analysis vaccination is assumed to begin 15 days after the epidemic start. Alternative timing was explored as sensitivity analyses, i.e., vaccination is introduced 30 days after (***SE2***), or 30 days before (***SE3***) the epidemic start.

The model is run considering daily time steps. Gradual delivery of vaccine doses is implemented by vaccinating a fixed number of individuals each day. Although manufacturers state that a total of 21 billion doses of vaccines could be available by the end of 2021^43^, scale-up and delivery will take months. On the basis of the 2009 H1N1 influenza pandemic vaccination program implemented in mainland China^14^, in the main analysis we assumed 6 million doses of COVID-19 vaccines could be administered each day (4 doses per 1,000 individuals) until uptake reaches 70% for all groups^44^. Different values of the daily vaccine administration capacity, i.e., 1.3 (***SE4***), 10 (***SE5***), 15 (***SE6***), and 30 (***SE7***) million dose per day, and different coverage levels, i.e. 50% (***SE8***) and 90% (***SE9***)^44^, are explored in separate sensitivity analyses.

In the main analysis, vaccination is administered to susceptible individuals only. This represents an ideal scenario where we assume that all infected individuals can be identified (e.g., either via RT-PCR while infected or via serological assays later one) and that SARS-CoV-2 infection confers a long-lasting immunity. Since infection ascertainment could be challenging and pose additional strain to the health system, we also consider two sensitivity analyses in which only detected symptomatic cases are excluded from vaccination (***SE10- SE11***).

In the context of fast RT-PCR–based mass screening if there is an outbreak, under-ascertainment of symptomatic cases could be only related with the sensitivity of RT-PCR tests. The sensitivity is quite high (98%) if the interval between symptom onset and RT-PCR test is within 7 days, and the sensitivity decreases to 68% if the time interval is 8–14 days^45^. The mean time interval from symptom onset to the date of collection of the sample for PCR testing was estimated to be 4.7 days in Hunan^36^. Accordingly, we considered as ascertainment probabilities of symptomatic cases 70% (***SE10***) and 90% (***SE11***).

#### Vaccination schedule and efficacy

There are six COVID-19 vaccines developed by China in phase 3 clinical trials, including five vaccines administered with a two-dose schedule with an interval of 14, 21, or 28 days and one single-dose recombinant adenovirus type-5-vectored vaccine. For simplicity, in the main analysis, we modeled the administration of an inactivated vaccine developed by the Beijing Institute of Biological Products,^46^ which entail a two-dose schedule across all age groups with an interval of 21 days. In a separate sensitivity analysis, we explored an interval of 14 days (***SE12***).

China approved its first local COVID-19 vaccine (developed by Sinopharm) for general public use on December 31, 2020, with an estimated vaccine efficacy (VE) of 79.3%.^28^ In the main analysis, we used a VE of 80% against infection in individuals aged 20–59 years. In the developed model, vaccination confers a partial protection, i.e., vaccinated individuals are 80% less likely to develop infection upon an infectious contact. Sensitivity analyses using a VE of 60% (***SE13***) and 90% (***SE14***) were separately performed. The alternative values of VE were selected on the basis of published upper efficacy of vaccines of 94–95% and in such a way to cover a plausible efficacy range of forthcoming vaccines.^29–31^

Phase 2 clinical trials demonstrated that vaccine immunogenicity was lower among older individuals than in younger adults^46^. And for other inactivated vaccines like influenza vaccine, a lower VE is observed in children compared to young adults^47^. Accordingly, we assumed an age-dependent VE. In particular, given a baseline efficacy VE among individuals aged 20–59 years (80% in the main analysis), we assumed a 50% lower VE in individuals <20 and ≥60 years of age (namely 40%). A scenario without age-specific variations in VE was explored as sensitivity analysis (***SE15***).

Individuals vaccinated with the first dose could still develop infections without any immune protection, while the second dose vaccination could produce the expected vaccine efficacy after an average of 14 days. In the main analysis we assume both natural infection-induced and vaccine-induced immunity to SARS-CoV-2 infection does not wane within the considered time horizon (2 years). In additional sensitivity analyses, we considered an average duration of vaccine-induced protection of 6 months (***SE16***) and 1 year (***SE17***). We also consider a sensitivity analysis assuming that vaccination is effective in preventing symptomatic illness, but not infection (***SE18***), and another one assuming an all-or-nothing vaccine, i.e., the vaccine confers full protection to VE percent of vaccinated individuals (***SE19***).

#### Priority order of vaccination

The doses available to be distributed daily (6 million in the main analysis) are assigned by considering the following order of priority^35^. In the main analysis, healthcare workers are considered as the top priority (Tier 1 of the vaccination strategy); law enforcement and security workers, personnel in nursing home and social welfare institutes, community workers, workers in energy, food and transportation sectors are included in Tier 2; adults ≥ 60 years of age with underlying conditions, and adults ≥ 80 years of age without underlying conditions, who are at the highest risk of severe/fatal COVID-19, are considered in Tier 3; individuals aged < 60 years with pre-existing medical conditions and pregnant women are included in Tier 4; individuals aged 20–59 years without underlying conditions are included in Tier 5; school-age children and younger children aged ≤5 years without underlying conditions are recommended for vaccination in Tier 6 (Supplementary File 3).

Different priority orders are explored as sensitivity analyses. Healthcare workers and the other essential workers listed above are fixed in Tier 1 and Tier 2 of vaccination, while the remaining population is vaccinated as described in Table 2 by considering different orders of prioritization only based on age and disregarding the presence of underlying conditions (***SE20***: first prioritization to old adults; ***SE21***: first prioritization to working-age groups***; SE22***: first prioritization to school-age groups). We explore the impact of 5,000 initial cases on the prioritization strategy (***SE23***). To understand the impact in terms of number of infections by age, we compare the prioritization strategy when we account for the uncertainty in the contact matrix and in the susceptibility to infection by age, or not (in this context, median values of contact numbers and relative susceptibility are used).

### COVID-19 burden model

The main output of above transmission model is the age-specific number of new infections per day in the subpopulation with or without underlying conditions. On top of that, we developed a model of COVID-19 disease burden to estimate the number of symptomatic cases, hospitalization, ICU admissions, and deaths in different scenarios in the presence/absence of vaccination.

We computed the age-specific number of symptomatic infections in individuals with and without underlying conditions on a daily-basis, by applying an age-specific probability of respiratory symptoms, which is 18.1%, 22.4%, 30.5%, 35.5%, and 64.6% separately for 0–19, 20–39, 40–59, 60–79, and 80+ years of age, as estimated from contact tracing data in Lombardy^8^. We assume that individuals with and without underlying conditions have the same age-specific probability of developing symptoms.

The daily age-specific number of hospital admissions in the two subpopulations was computed by applying the age-specific proportion of laboratory-confirmed symptomatic cases requiring hospitalizations (Supplementary File 4), delayed by an average time of 3.8 days between symptom onset and hospitalization.^11^

The daily age-specific number of patients admitted to ICU in the two subpopulations was computed by applying to hospitalized cases an age-specific probability of being admitted to ICU^13^, and distinguishing patients requiring intensive care in survivors and non-survivors. Survivors are admitted to ICU after an average time of 7 days from hospitalization. Non-survivors are admitted to ICU after an average time of 8 days after hospitalization^10^.

The daily age-specific number of deaths in the two subpopulations was computed by applying the age-specific fatality ratio among symptomatic cases (Supplementary File 4), delayed by an average time of 13.9 days between symptom onset and death.^12^

### Data analysis

For each scenario, 200 stochastic model realizations were performed. The outcome of these simulations determined the distributions of the number of symptomatic infections, hospitalizations, ICU admissions, and deaths. 95% confidence intervals were defined as quantiles 0.025 and 0.975 of the estimated distributions. We used a Bayesian approach to estimate R_t_ from the time series of symptomatic cases by date of symptom onset and the distribution of the serial interval^11^. The methods were described in Supplementary File 5 in details.

## Supplementary Material

Supplement

Supplement
