# Supplementary material for: Can a COVID-19 vaccination program guarantee the return to a pre-pandemic lifestyle?
Source: Res Sq. 2021 Feb 9:rs.3.rs-200069. Preprint. [Version 1] doi: 10.21203/rs.3.rs-200069/v1 (PMC7885929; doi:10.21203/rs.3.rs-200069/v1)
Supplement: Supplement [file 5c90de8cbe8c62ffea260d14.docx]

**Extended Data**


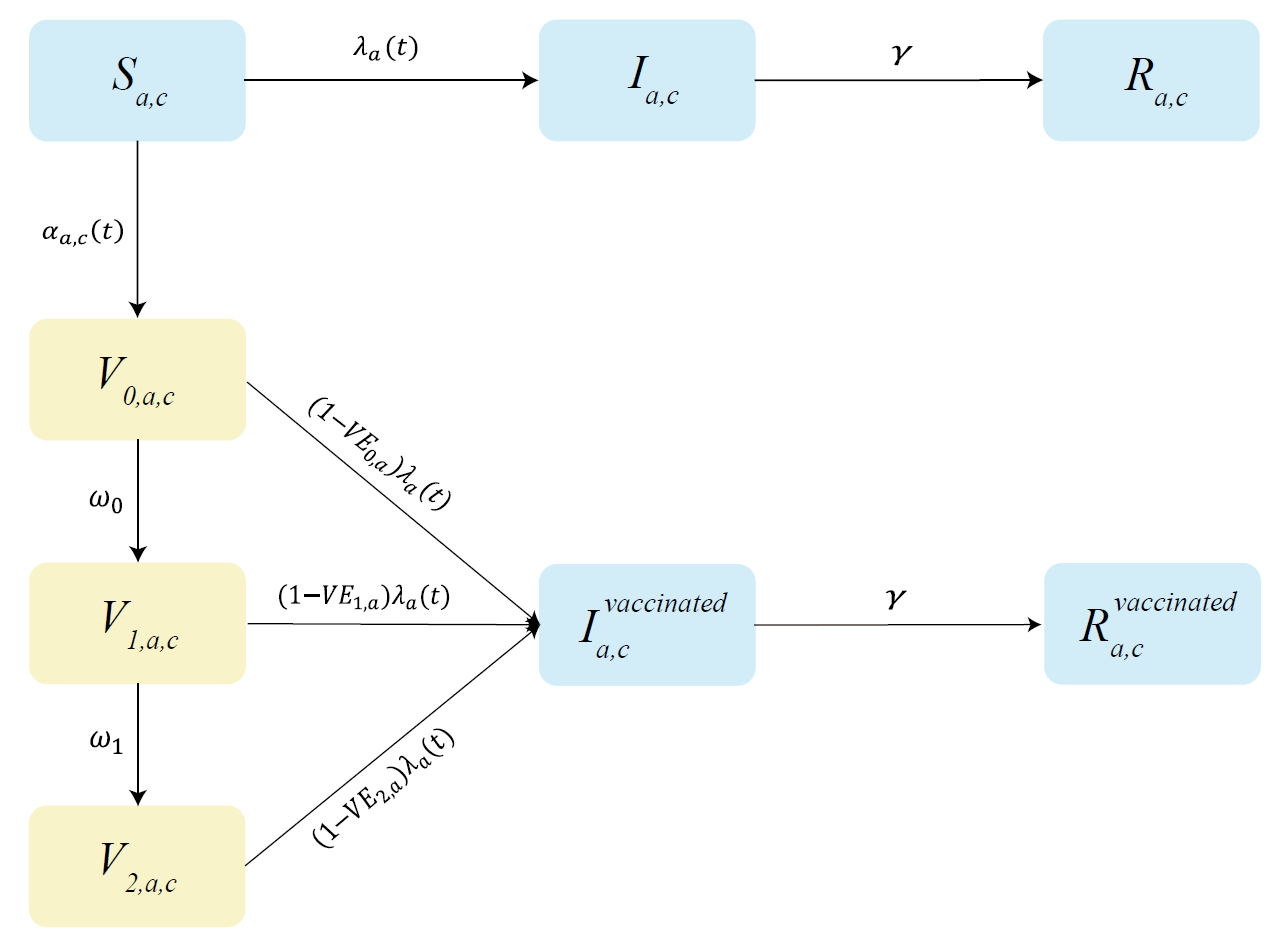


**Extended Data Fig. 1 Schematic representation of the baseline model.**

Blue, and yellow rectangles describe the SARS-CoV-2 transmission model, and vaccination model, respectively. Transitions occur within each population class defined by a and c, where a represents the age group and c identifies the absence/presence of underlying conditions, the latter associated with higher risk of sever outcomes of SARS-CoV-2 infections. S denotes susceptible individuals; I infected individuals; R recovered/removed individuals. Parameters of the transmission model include$:$the time- and age-dependent force of infection$\lambda_{a}\left( t \right)$ and the recovery rate from infection ($\gamma)$. V_0_ denotes individuals vaccinated with the 1^st^ dose (never experienced infection with SARS-CoV-2); V_1_ denotes vaccinated with the 2^nd^ dose (no protection yet); V_2_ denotes vaccinated with the 2^nd^ dose (protected). Parameters of the vaccination model include: the time-, age- and group-dependent probability of being vaccinated $(\alpha_{a,c}\left( t \right))$; the interval between administration of the first and second dose (1/$\omega_{0}$); the delay of ramp-up of the 2^nd^ dose (${1/\omega}_{1}$); the age-dependent vaccine efficacy after the 1^st^ dose of vaccination (${VE}_{0,a})$, which is assumed to be 0; the age-dependent efficacy right after administration of the 2^nd^ dose (${VE}_{1,a}),$which is assumed to be 0; the vaccine efficacy after ramp-up of the 2^nd^ dose (${VE}_{2,a})$.

**Extended Data Table 1. Description of key parameters used in the model.**

| **Description of parameter** | **Values in the baseline analysis** | **Sensitivity analyses** |
| --- | --- | --- |
| **Epidemiology** |  |  |
| Generation time (1/$\gamma)$ | 5.5 days (95%CI 1.7, 11.6)^34^ | / |
| Relative susceptibility to infection at age $a$ ($r_{a}$) | $r_{a}=0.58$ (95%CI 0.34-0.98) when $a<15$; $r_{a}=1$ for $15\leq a<65$; $r_{a}=1.65$ (95%CI 1.03-2.65) when $a\geq65$ ^34^ | Homogenous susceptibility *(****SE1****)* |
| Age-group-specific contact matrix ($C_{a,\tilde{a}}$) | Contact matrix for Shanghai ^7^ | / |
| Effective reproductive number (R_t_) | 1.1, 1.3, 1.5, and 2.5 ^11,35,36^ | / |
| **Vaccination** |  |  |
| Interval between the administration of 1^st^ dose and 2^nd^ dose (1/$\omega_{0}$) | 21 days ^37^ | 14 days *(****SE12****)* ^37^ |
| Delay between administration of the 2^nd^ dose of vaccination and the achievement of the expected VE (1/$\omega_{1}$) | 14 days ^37^ | / |
| Expected vaccine efficacy for adults aged 20-59 years (${VE}_{2,a}$) | 80% ^37,38^ for a vaccine with partial protections | 60% *(****SE13****)* and 90% *(****SE14****);* and for an all-or-nothing vaccine *(****SE19****)* |
| Expected vaccine efficacy reduction for <20 and ≥60 years | 50% ^37,38^ | 0%, indicating the same vaccine efficacy *(****SE15****)* |
| Vaccination capacity (daily doses administered) | 6 million (Assumed based on 2009 influenza pandemic vaccination) ^22^ | 1.3 *(****SE4****)*, 10 *(****SE5****)*, 15 *(****SE6****)* and 30 million *(****SE7****)* |
| Vaccine coverage | 70% ^39^ | 50% (***SE8***) or 90% *(****SE9****) ^39^* |
| Duration of immunity (1/$\omega_{2}$) | Lifelong (i.e., the immunity lasts more than the time horizon considered: 730 days)  (Assumed) | 6 months *(****SE16****)*, or 1 year *(****SE17****)* |
| Delay between start of simulations and start of vaccination | 15 days (Assumed) | 30 days prior- *(****SE2****)* or post-start *(****SE3****)* of simulations |
| **Disease burden** |  |  |
| Proportion of infections that develop symptoms ($\pi$) | 18.1%, 22.4%, 30.5%, 35.5%, and 64.6% separately for 0-19, 20-39, 40-59, 60-79, and 80+ years^8^ | / |
| Proportion of laboratory-confirmed symptomatic cases requiring hospitalization ($\sigma$) | Overall: 40.0%, 29.2%, 33.3%, and 33.8% separately for 0-19, 20-39, 40-59, and 60+ years^9^; estimates for individuals with/without underlying conditions shown in Supplementary Information File 1 | / |
| Proportion of hospitalized cases requiring ICU ($\rho$) | 0, 2.2%, 7.2%, 20.9% separately for 0-14, 15-49, 50-64, and 65+ years^13^ | / |
| Fatality ratio among laboratory-confirmed symptomatic cases ($\mu$) | Overall: 0.51%, 0.65%, 2.38%, and 10.52% separately for 0-19, 20-39, 40-59, and 60+ years^9^; estimates for individuals with/without underlying conditions shown in Supplementary Information File 1 | / |


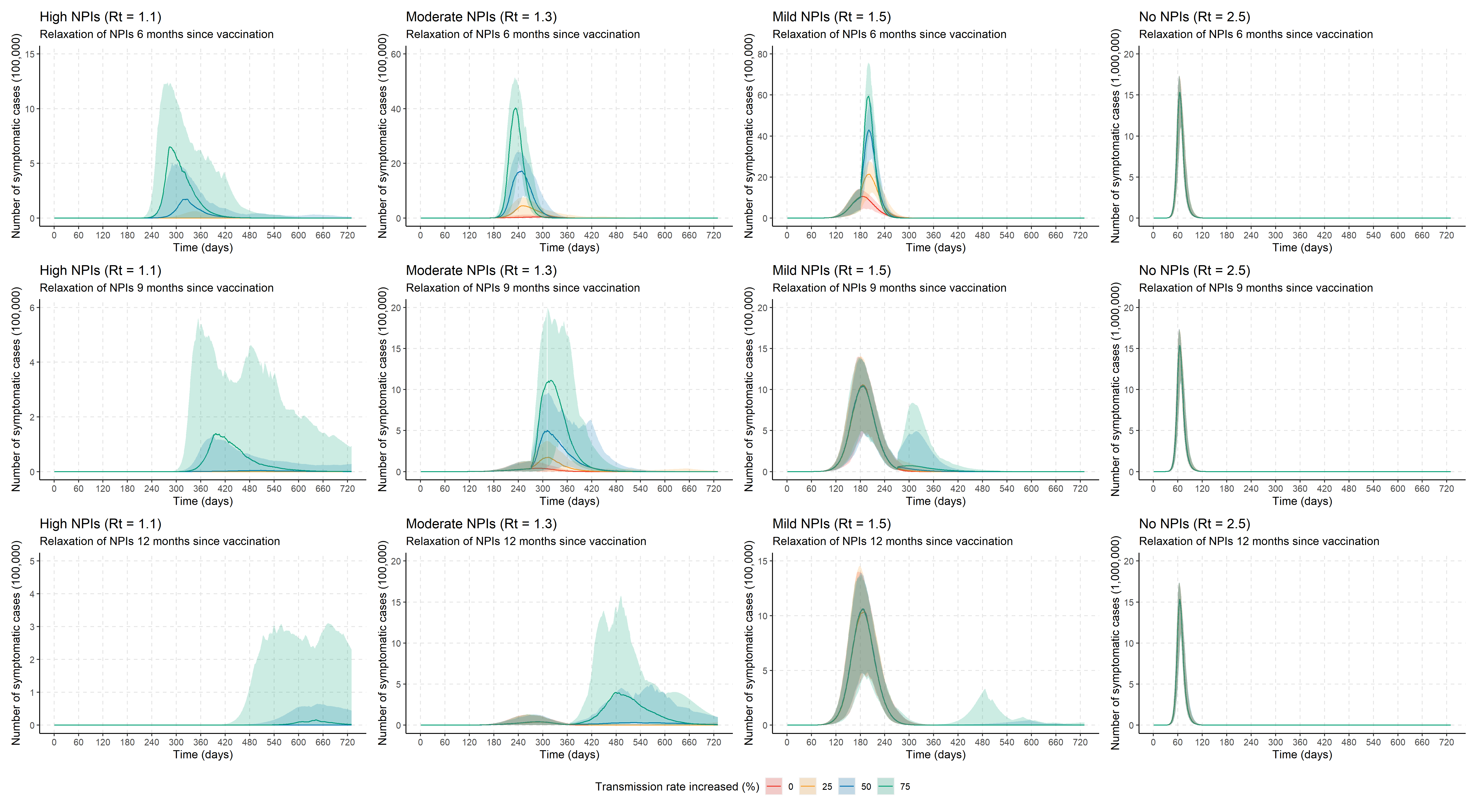


**Extended Data Figure.2 Time series of symptomatic cases given relaxation of NPIs 6, 9 and 12 months since vaccination.**

We assumed a 0% (i.e., baseline analysis, no relaxation of NPIs during study periods), 25%, 50% and 75% increase of transmission rate, respectively. Line denotes median, and shadow denotes quantiles 0.025 and 0.975.


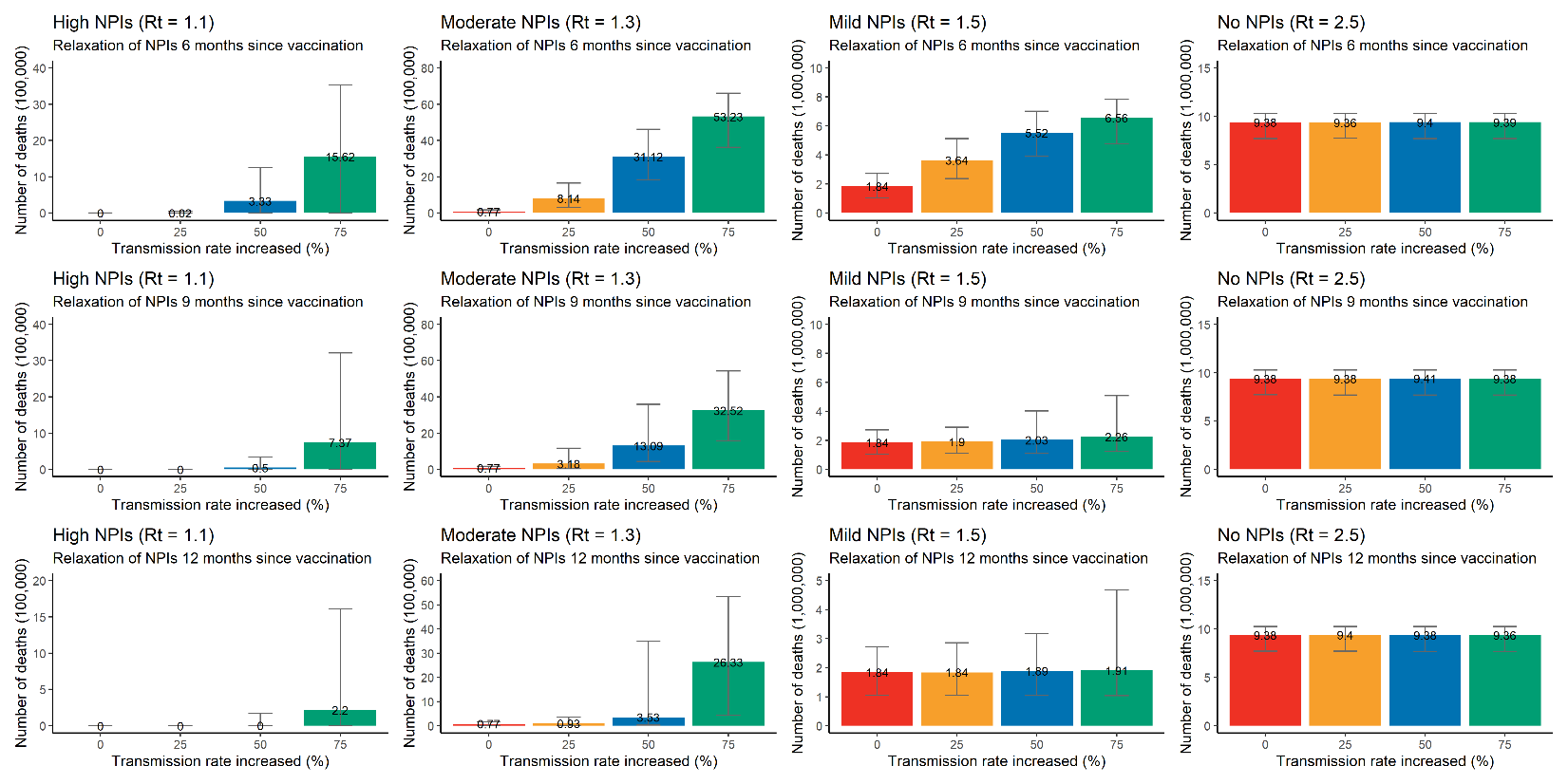


**Extended Data Figure.3 Cumulative number of COVID-19 deaths given relaxation of NPIs 6, 9 and 12 months since vaccination.**

We assumed a 0% (i.e., baseline analysis, no relaxation of NPIs during study periods), 25%, 50% and 75% increase of transmission rate, respectively. Number denotes median, and error bars denote quantiles 0.025 and 0.975.


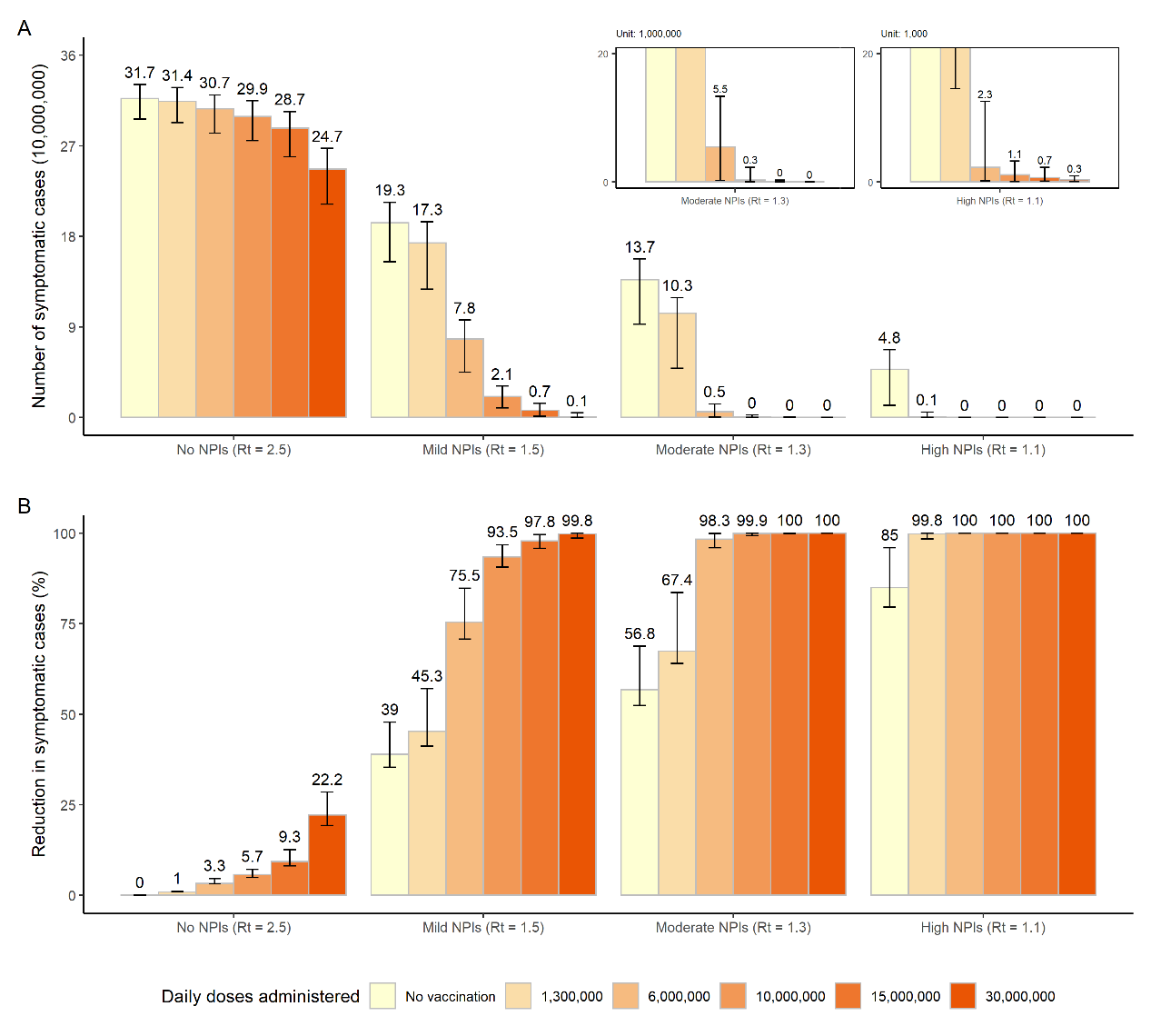


**Extended Data Figure.4 Impact of daily doses administered on COVID-19 symptomatic cases.**

A) Cumulative number of COVID-19 symptomatic cases as estimated in the different scenarios under progressively increasing values of the daily vaccination capacity; B) Proportion of symptomatic cases averted compared to the *reference scenario*, i.e., no vaccination + no NPIs with initial R_t_=2.5. Number denotes median, and error bars denote quantiles 0.025 and 0.975.


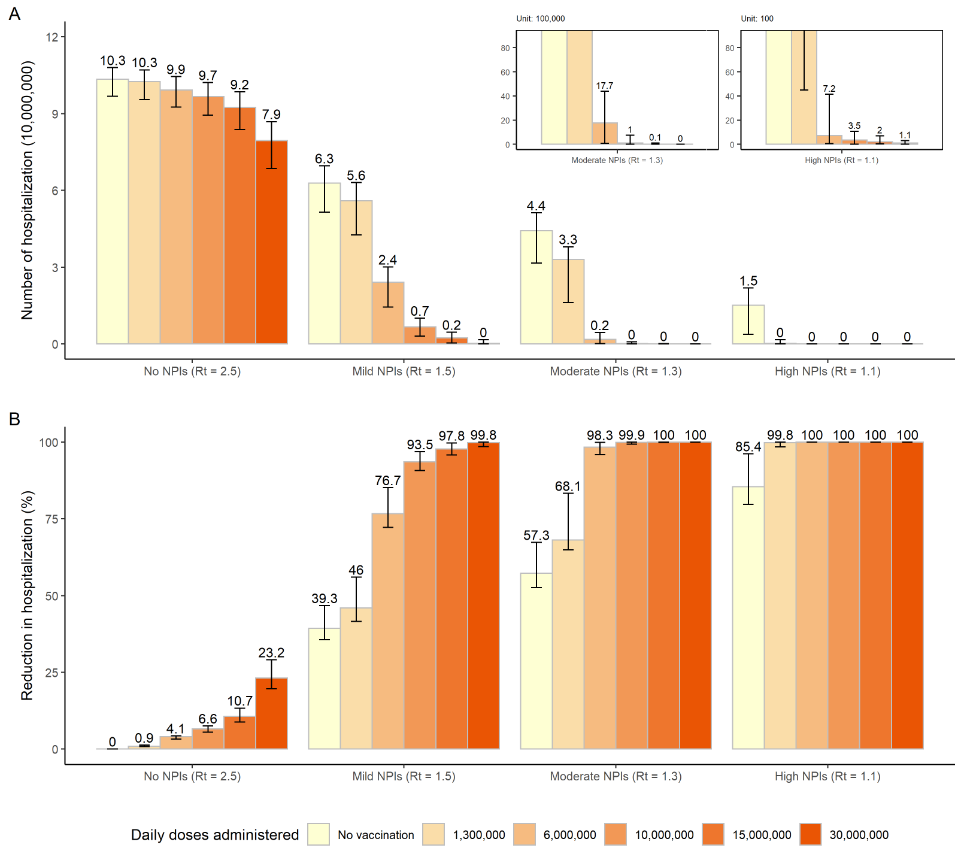


**Extended Data Figure.5 Impact of daily doses administered on COVID-19 hospitalizations.**

A) Cumulative number of COVID-19 hospitalizations as estimated in the different scenarios under progressively increasing values of the daily vaccination capacity; B) Proportion of hospitalizations averted compared to the *reference scenario*, i.e., no vaccination + no NPIs with initial R_t_=2.5. Number denotes median, and error bars denote quantiles 0.025 and 0.975.


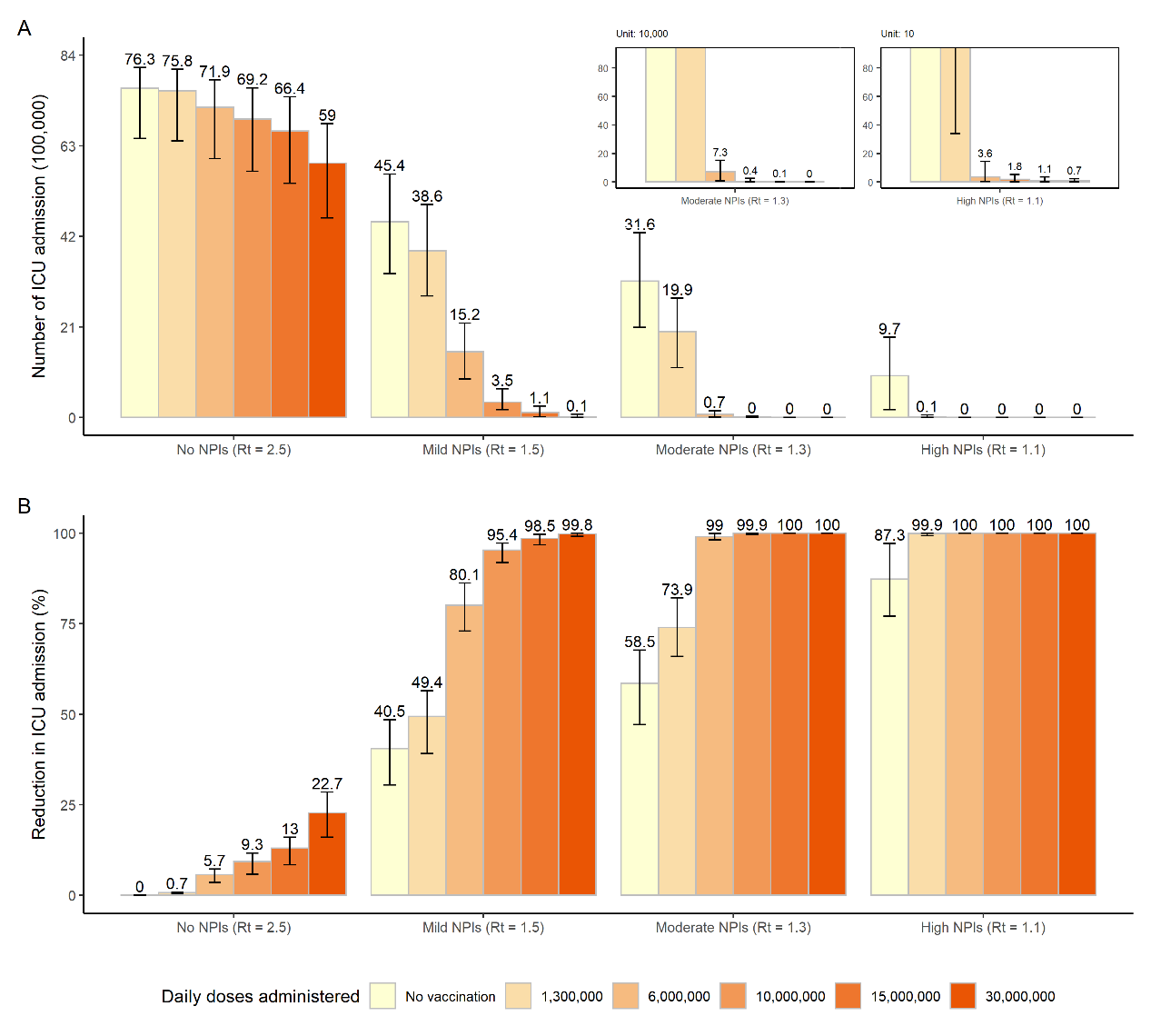


**Extended Data Figure.6 Impact of daily doses administered on COVID-19 ICU admissions.**

A) Cumulative number of COVID-19 ICU admissions as estimated in the different scenarios under progressively increasing values of the daily vaccination capacity; B) Proportion of ICU admissions averted compared to the *reference scenario*, i.e., no vaccination + no NPIs with initial R_t_=2.5. Number denotes median, and error bars denote quantiles 0.025 and 0.975.


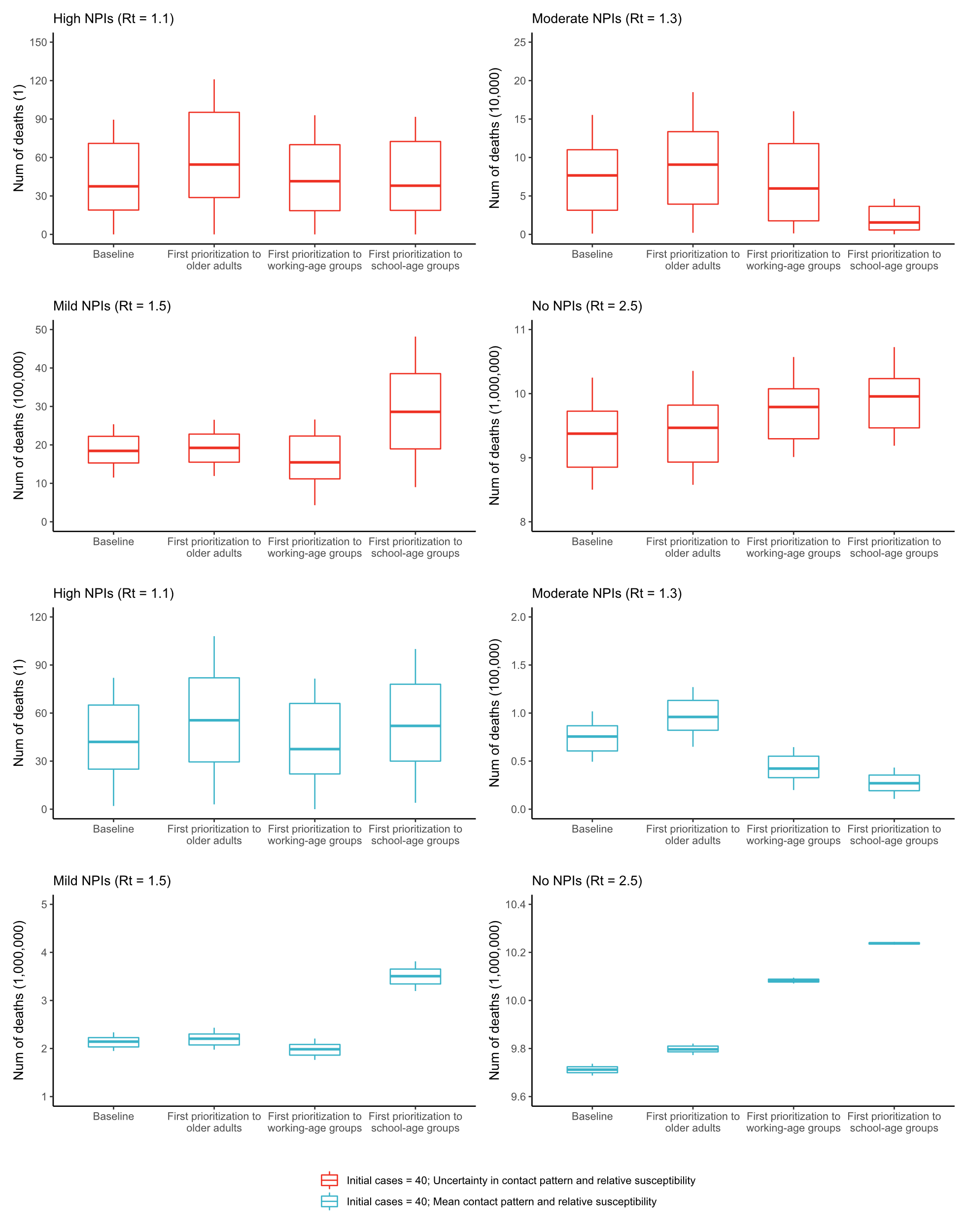


**Extended Data Figure.7 Overall impact of vaccination prioritizations on cumulative COVID-19 deaths provided 6 million doses administered/day and 40 initial cases.**

The baseline scenario means first prioritizing older adults and individuals with underlying conditions.


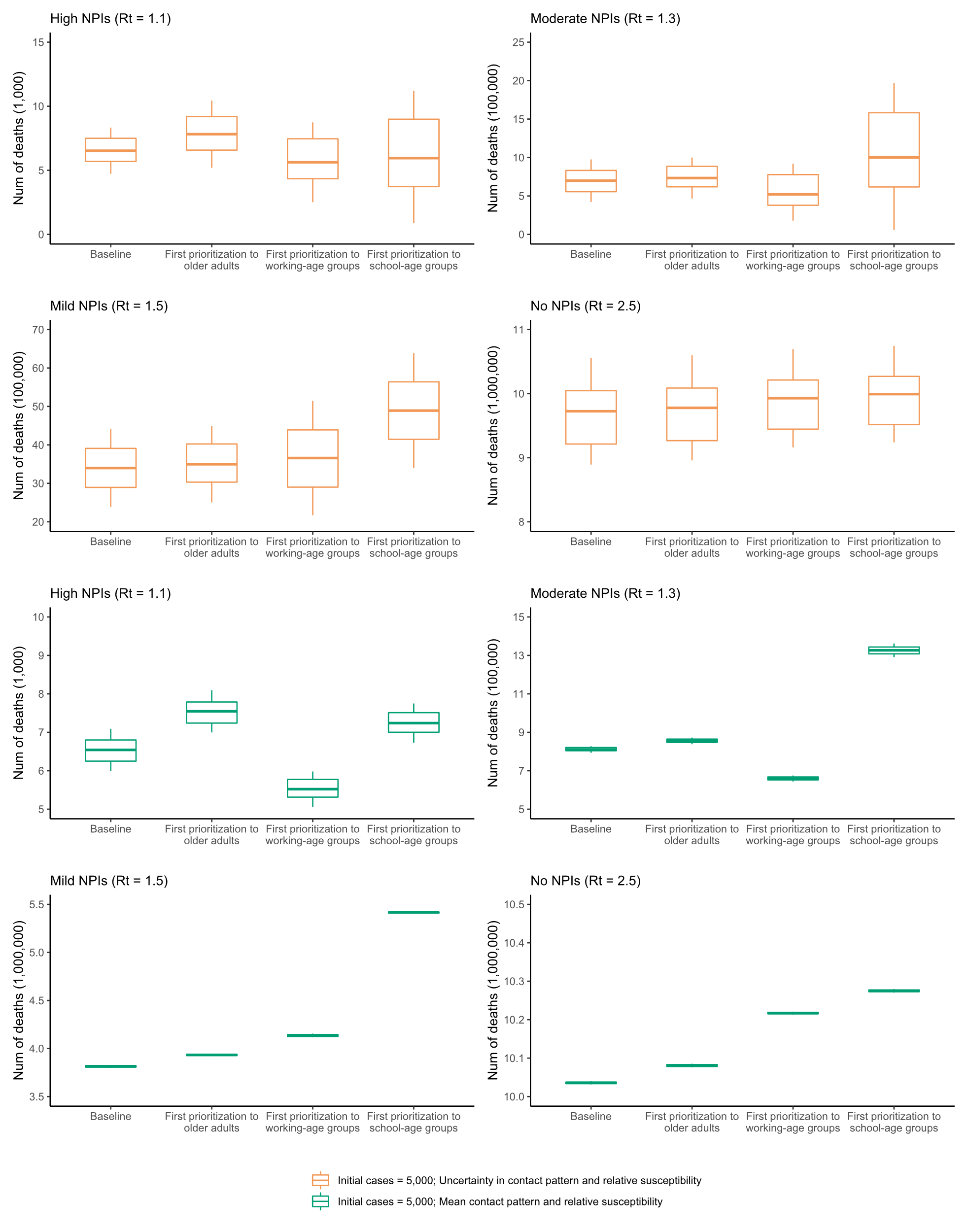


**Extended Data Figure.8 Overall impact of vaccination prioritizations on cumulative COVID-19 deaths provided 6 million doses administered/day and 5,000 initial cases.**

The baseline scenario means first prioritizing older adults and individuals with underlying conditions.


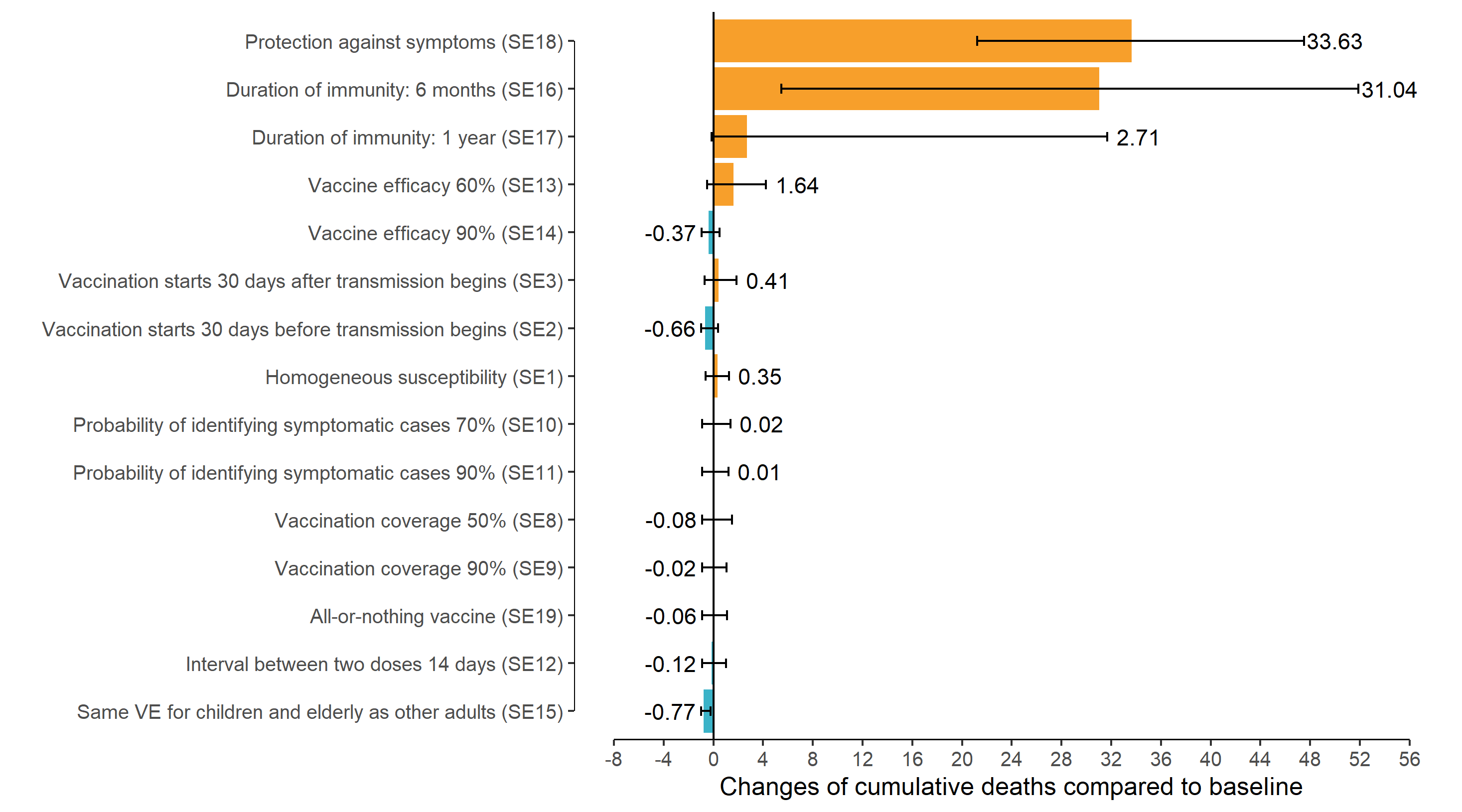


**Extended Data Figure.9** **Changes of the cumulative number of COVID-19 deaths estimated in the different sensitivity analyses, compared to the main analysis in the presence of moderate NPIs (R_t_=1.3)****.**

Number denotes median, and error bars denote quantiles 0.025 and 0.975.


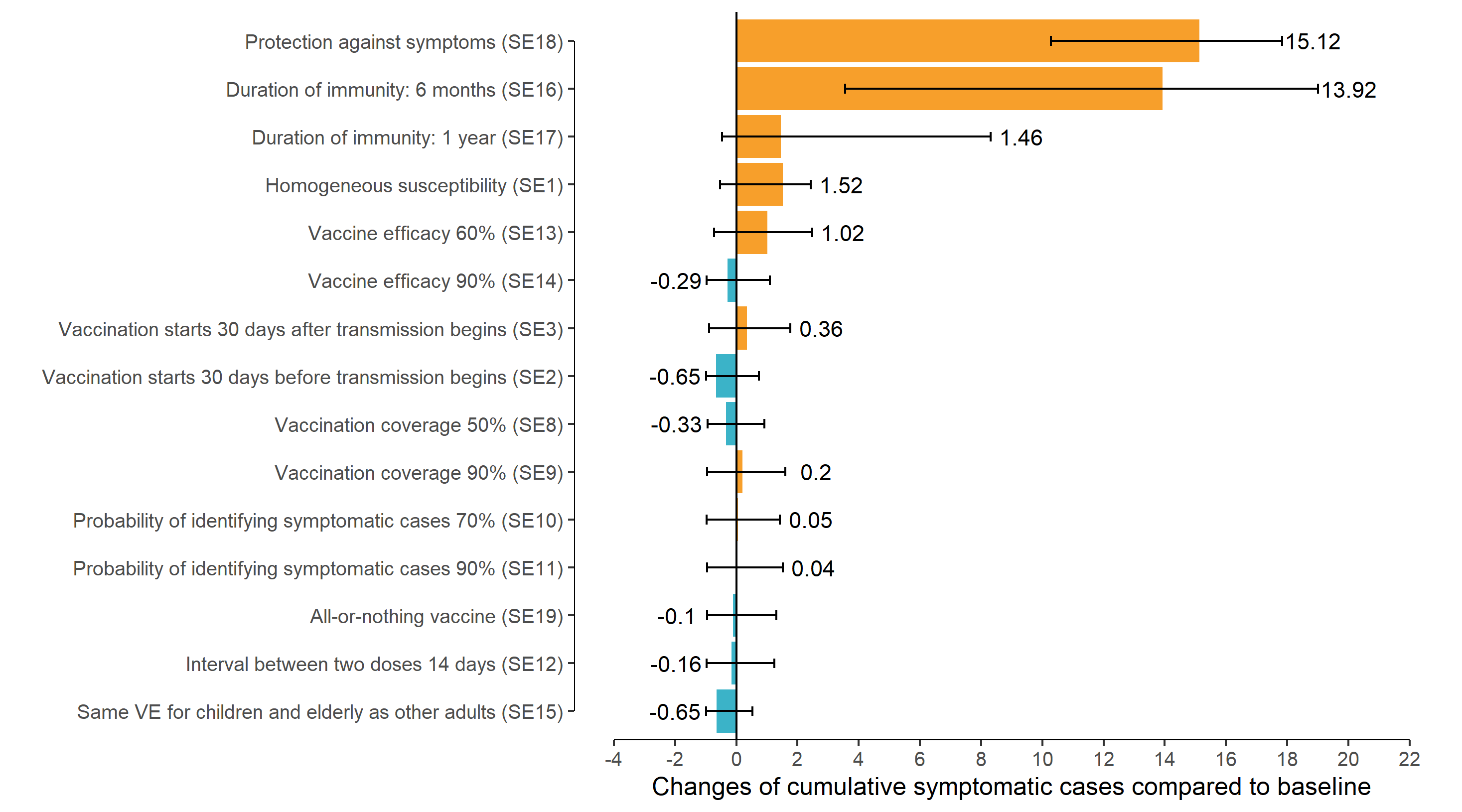


**Extended Data Figure.10 Changes of the cumulative number of COVID-19 symptomatic cases estimated in the different sensitivity analyses and in the main analysis in the presence of moderate NPIs (initial Rt=1.3)**

SE: sensitivity analysis. Number denotes median, and error bars denote quantiles 0.025 and 0.975.
