# Supplementary material for: Can a COVID-19 vaccination program guarantee the return to a pre-pandemic lifestyle?
Source: Res Sq. 2021 Feb 9:rs.3.rs-200069. Preprint. [Version 1] doi: 10.21203/rs.3.rs-200069/v1 (PMC7885929; doi:10.21203/rs.3.rs-200069/v1)
Supplement: Supplement [file c3d85017c941b681767f6f5f.docx]

Supplementary Information

**Can a COVID-19 vaccination program guarantee the return to a pre-pandemic lifestyle?**

Juan Yang^1,^*, Valentina Marziano^2,^*, Xiaowei Deng^1^, Giorgio Guzzetta^2^, Juanjuan Zhang^1^, Filippo Trentini^2^, Jun Cai^1^, Piero Poletti^2^, Wen Zheng^1^, Wei Wang^1^, Qianhui Wu^1^, Zeyao Zhao^1^, Kaige Dong^1^, Guangjie Zhong^1^, Cécile Viboud^3^, Stefano Merler^2,†^, Marco Ajelli^4,5,†^, Hongjie Yu^1,6,7,†^

1. School of Public Health, Fudan University, Key Laboratory of Public Health Safety, Ministry of Education, Shanghai, China
2. Bruno Kessler Foundation, Trento, Italy
3. Division of International Epidemiology and Population Studies, Fogarty International Center, National Institutes of Health, Bethesda, MD, USA
4. Department of Epidemiology and Biostatistics, Indiana University School of Public Health, Bloomington, IN, USA
5. Laboratory for the Modeling of Biological and Socio-technical Systems, Northeastern University, Boston, MA, USA
6. Shanghai Institute of Infectious Disease and Biosecurity, Fudan University, Shanghai, China
7. Department of infectious diseases, Huashan Hospital, Fudan University Shanghai, China

*These authors contributed equally to this work.

^†^These authors are joint senior authors contributed equally to this work.

Corresponding authors: Marco Ajelli, Department of Epidemiology and Biostatistics, Indiana University School of Public Health, 47405 Bloomington, IN, USA; E-mail: marco.ajelli@gmail.com, and Hongjie Yu, Fudan University, School of Public Health, Key Laboratory of Public Health Safety, Ministry of Education, Shanghai 200032, China; E-mail: yhj@fudan.edu.cn

Contents

[Supplementary file 1. SARS-CoV-2 transmission and vaccination models 4](#_Toc63238631)

[Supplementary file 2. Estimating of the scaling factor for transmissibility in the absence of NPIs ($\boldsymbol{\beta}$) 8](#_Toc63238632)

[Supplementary file 3. Priority population of COVID-19 vaccination 9](#_Toc63238633)

[Supplementary file 4. Estimating the proportion of laboratory-confirmed COVID-19 symptomatic cases requiring hospitalization and death for individuals with and without underlying conditions 10](#_Toc63238634)

[Supplementary file 5. Data analysis 13](#_Toc63238635)

[References 15](#_Toc63238636)

# Supplementary file 1. SARS-CoV-2 transmission and vaccination models

We developed a model of SARS-CoV-2 transmission and vaccination, based on an age-structured stochastic susceptible-infectious-removed (SIR) scheme, accounting for heterogeneous mixing patterns by age as estimated in Shanghai ^1^. The Chinese population was distributed in 18 age groups (17 5-year age groups from 0 to 84 years and one age group for individuals aged 85 years or older) ^2^. Each age group was further split into two subgroups: individuals with or without underlying conditions, where the former were considered to be associated with an increased risk of severe outcome of COVID-19 ^3^.

In the main analysis, susceptibility to SARS-CoV-2 infection was assumed to be heterogeneous across ages. Children under 15 years of age were considered less susceptible to infection compared to adults aged 15 to 65 years, while the elderly more susceptible ^4^. Asymptomatic and symptomatic individuals were assumed to be equally infectious ^4,5^, and infectiousness was also assumed to be the same across age groups ^4,5^.

Vaccine is administered with a two-dose schedule. In the baseline model, we assumed that: i) vaccination reduces susceptibility to SARS-CoV-2 infection; ii) only susceptible individuals are eligible for vaccination, i.e., we excluded all individuals that have experienced SARS-CoV-2 infection; iii) duration of vaccine-induced protection lasts longer than the time horizon considered (2 years).

The baseline model is schematically represented in Extended Data Fig. 1 and it is described by the following differential systems:

$$\left\{ \begin{aligned} S_{a,c}^{'}\text{(t)= }{-\lambda}_{a}\left( t \right)S_{a,c}\left( t \right)-\alpha_{a,c}\left( t \right)S_{a,c}\left( t \right) \\ I_{a,c}^{'}\text{(t)= }\lambda_{a}\left( t \right)S_{a,c}\left( t \right)-\gamma I_{a,c}\left( t \right) \\ R_{a,c}^{'}\text{(t)= }\gamma I_{a,c}\left( t \right) \\ V_{0,a,c}^{'}\text{(t)=}\alpha_{a,c}\left( t \right)S_{a,c}\left( t \right)-\left( 1{-VE}_{0,a} \right)\lambda_{a}\left( t \right)V_{0,a,c}-{\omega_{0}V}_{0,a,c}\left( t \right) \\ V_{1,a,c}^{'}\text{(t)=}{\omega_{0}V}_{0,a,c}\left( t \right)-\left( 1{-VE}_{1,a} \right)\lambda_{a}\left( t \right)V_{1,a,c}-{\omega_{1}V}_{1,a,c}\left( t \right) \\ V_{2,a,c}^{'}\text{(t)=}{\omega_{1}V}_{1,a,c}\left( t \right)-\left( 1{-VE}_{2,a} \right)\lambda_{a}\left( t \right)V_{2,a,c} \\ I_{a,c}^{\mathrm{vaccinated} '}\text{(t)=}\lambda_{a}\left( t \right)[\left( 1{-VE}_{0,a} \right)V_{0,a,c}+\left( 1{-VE}_{1,a} \right)V_{1,a,c}+\left( 1{-VE}_{2,a} \right)V_{2,a,c}]-\gamma I_{a,c}^{\mathrm{vaccinated}}\text{(}\text{t}\text{)} \\ R_{a,c}^{\mathrm{vaccinated} '}\text{(t)=}\gamma I_{a,c}^{\mathrm{vaccinated}}\text{(}\text{t}\text{)} \end{aligned} \right.$$

where:

$S_{a,c}$ represents the number of susceptible to SARS-CoV-2 infection in the population class *{a,c}*, where *a* represents the age group and *c* identifies the absence/presence of underlying conditions.

$I_{a,c}$represents the number of infectious unvaccinated individuals in the population class *{a,c}.*

$R_{a,c}$represents the number of unvaccinated individuals in the population class *{a,c}* who recovered from infection.

$V_{0,a,c},V_{1,a,c},{and V}_{2,a,c}$ represent the number of vaccinated individuals in each ramp-up stage. In particular,

$V_{0,a,c}$denotes individuals in the population class *{a,c}* vaccinated with the first dose. In the main analysis, we assumed that the second dose is administered 21 days after the 1^st^ dose. So ${1/\omega}_{0}=21$ days.

$V_{1,a,c}$ denotes individuals in the population class *{a,c}* vaccinated with the second dose, for whom the 2^nd^ dose is not effective yet. We assumed that the second dose becomes effective 14 days after administration, so ${1/\omega}_{1}=14$ days.

$V_{2,a,c}$ denotes individuals in the population class *{a,c}* vaccinated with the second dose for whom vaccination is effective.

$I_{a,c}^{\mathrm{vaccinated}}$represents the number of infectious individuals in the population class *{a,c}* among those who have already received at least one dose of vaccination.

$R_{a,c}^{\mathrm{vaccinated}}$represents the number of individuals in the population class *{a,c}* who developed infection despite having received vaccination (one or more doses).

Susceptible individuals are exposed to a time and age-dependent force of infection$\lambda_{a}\left( t \right)$ which is defined as:

$$\lambda_{a}\left( t \right)=\left( 1-\varphi\right)\beta r_{a}\sum_{\tilde{a}} C_{a,\tilde{a}}\frac{\sum_{c} [I_{\tilde{a},c} \left( t \right)+I_{\tilde{a},c}^{vaccinated}\left( t \right)]}{\sum_{c} N_{\tilde{a},c}}$$

where:

$\beta$ is a scaling factor shaping SARS-CoV-2 transmissibility in the absence of non-pharmaceutical interventions (no NPIs, Effective reproductive number R_t_ =2.5), such as social distancing, school closure, and case isolation.

$\varphi$ is a coefficient representing the reduction in transmissibility due to NPIs.

$r_{a}$is the relative susceptibility to SARS-CoV-2 infection at age $a$: $r_{a}=0.58$ (95%CI 0.34-0.98) when $a<15$; $r_{a}=1$ for $15\leq a<65$*;* $r_{a}=1.65$ (95%CI 1.03-2.65) when $a\geq65$ ^4,5^.

$C_{a,\tilde{a}}$ represents the age-group-specific contact matrix, whose entries describe the mean numbers of persons in age group $\tilde{a}$ encountered by an individual of age group $a$ per day.

$N_{\tilde{a},c}$ represents the number of individuals in the population class *{*$\tilde{a}$*,c}*.

For all infectious compartments, the average duration of infectiousness $(1/\gamma)$ is set equal to the average generation time (5.5 days)^4^.

At each time *t*, the first dose of vaccination is administered to a fraction $\alpha_{a,c}\left( t \right)$of susceptible individuals in the population class *{a,c}*:

$$\alpha_{a,c}\left( t \right)=\frac{d_{a,c}(t)}{S_{a,c}\left( t \right)}$$

where $c$ represents the number of (first) vaccine doses to be administered to individuals of the population class *{a,c}* at time *t* under the considered vaccination scenario.

The daily number of first doses $d_{a,c}\left( t \right)$ to be administered to the population class *{a,c}* is computed by taking into account: i) the assumed priority order; ii) the assumed vaccination coverage, i.e. the fraction of population that is expected to be vaccinated at the end of the program; ii) the constraints on the daily vaccination capacity. In particular, we assume that half of the daily capacity is allocated to first doses, i.e.:

$\sum_{a,c} d_{a,c}\left( t \right)$= (daily vaccination capacity)/2

and the remaining half to second doses.

Vaccinated individuals $V_{i,a,c} \left( \text{i=0,1,2} \right)$can develop infection, but their susceptibility to infection is reduced by a factor ($1-{VE}_{i,a})$,where ${VE}_{i,a}$represents the age-specific vaccine efficacy associated to the *i*-th vaccination stage. In the main analysis, the age-dependent vaccine efficacy after the 1^st^ dose of vaccination (${VE}_{0,a})$was assumed to be 0; the age-dependent efficacy right after administration of the 2^nd^ dose (${VE}_{1,a})$was also assumed to be 0; while the vaccine efficacy after ramp-up of the 2^nd^ dose (${VE}_{2,a})$ was assumed to be 80% for individuals aged 20-59 years and 40% for all other age groups. Simulation results discussed in the main text and in the following sections were obtained by using a stochastic version of the model described above.

# Supplementary file 2. Estimating of the scaling factor for transmissibility in the absence of NPIs ($\boldsymbol{\beta}$)

The reproduction number can be computed as the dominant eigenvalue of the Next Generation Matrix (NGM) ^24^ associated with the dynamical system considered:

$${(NGM)}_{a,\tilde{a}}=\frac{\beta}{\gamma}r_{a}C_{a,\tilde{a}}$$

We assumed a reproduction number in the absence of NPIs $R_{t}\left( \text{no}\text{ }\text{NPIs} \right)=2.5$ (4,8,9). Given the value of $R_{t}\left( \text{no}\text{ }\text{NPIs} \right)$, the distribution of the age-specific susceptibility profile ($r_{a})$and the distribution of the bootstrapped contact matrix, we computed the distribution of $\beta$ analytically.

When considering a set of NPIs that are capable to bring the reproduction number to a value $R_{t}(\text{NPIs})$< $R_{t}(\text{no}\text{ }\text{NPIs})$, we used the distribution of $\beta$ obtained in the absence on NPIs, rescaled by a factor ($1-\varphi)$ where

$\varphi=1-R_{t}(\text{NPIs})$/$R_{t}(\text{no}\text{ }\text{NPIs})$.

# Supplementary file 3. Priority population of COVID-19 vaccination

**Table S1. Priority population of COVID-19 vaccination***

| Tier of vaccination | Baseline (First prioritization to old adults and individuals with underlying conditions) | First prioritization to old adults  (***SE20^ǂ^***) | First prioritization to working-age groups (***SE21***) | First prioritization to school-age groups (***SE22***) |
| --- | --- | --- | --- | --- |
| 1 | Healthcare workers (No=10.7 million) | | | |
| 2 | Law enforcement and security workers, personnel in nursing home and social welfare institutes, community workers, workers in energy, food and transportation sectors, etc.  (No=36.8 million) | | | |
| 3 | Adults ≥ 60 years of age with underlying conditions, and adults ≥ 80 years of age without underlying conditions  (No.=162.9 million) | Adults ≥ 60 years of age  (No= 248.6 million) | Individuals aged 20-59 years  (No= 807.2 million) | School-age children  (No= 237.4 million) |
| 4 | Older adults aged 60-79 years without underlying conditions, individuals aged < 60 years with pre-existing medical conditions, and pregnant women  (No.=401.2 million) | Individuals aged 20-59 years  (No= 807.2 million) | School-age children  (No= 237.4 million) | Individuals aged 20-59 years  (No= 807.2 million) |
| 5 | Individuals aged 20-59 years without underlying conditions  (No.=525.8 million) | School-age children  (No= 237.4 million) | Adults ≥ 60 years of age  (No= 248.6 million) | Adults ≥ 60 years of age  (No= 248.6 million) |
| 6 | School-age children and younger children ≤5 years  (No.=301.9 million) | Younger children≤5 years  (No= 98.7 million) | | |

**^*^**Healthcare workers and the other essential workers listed here are fixed in Tier 1 and Tier 2 of vaccination, and thus would be vaccinated before other subgroups. ^ǂ^Sensitivity analysis.

# Supplementary file 4. Estimating the proportion of laboratory-confirmed COVID-19 symptomatic cases requiring hospitalization and death for individuals with and without underlying conditions

In order to quantify the different burden of COVID-19 in individuals with and without underlying conditions (such as chronic respiratory disease, heart disease, cardio-cerebrovascular disease, hypertension, diabetes, chronic renal diseases, chronic liver disease, cancer, and obesity ^3^), we estimated the hospitalization and death rates for the two subgroups in China, using below data: 1) the overall age-specific hospitalization and death rates among symptomatic cases independent from the presence of underlying conditions in China ^29^; 2) the proportion of symptomatic cases hospitalized/died in the two subgroups as obtained from the Lombardy region of Italy ^25,30,31^.

The age-specific proportions of laboratory-confirmed symptomatic cases requiring hospitalization for individuals with ($\sigma_{a,u}$) and without ($\sigma_{a,nu}$) underlying conditions were computed respectively as:

$\sigma_{a,u} =s\cdot h_{u}^{ITA}\cdot$ $\Delta_{a}$

$\sigma_{a,nu} =s\cdot h_{nu}^{ITA}\cdot$ $\Delta_{a}$

Where,

- $h_{u}^{ITA}$ and$h_{nu}^{ITA}$ separately denote the proportion of hospitalized among symptomatic cases with and without underlying conditions as estimated from Lombardy data (Table S2) ^25,30,31^.
- $\Delta_{\mathbf{a}}$ denotes the age-specific proportion of laboratory-confirmed symptomatic cases requiring hospitalization as estimated for China independently from the presence of underlying conditions ^29^.
- the scale factor $s$ is determined in such a way to minimize the root mean square error between $\Delta_{a}$ and $\tilde{\Delta_{a}}=$ $P_{a,u}\cdot\sigma_{a,u} +P_{a,nu}\cdot\text{ }\sigma_{a,nu}.$ $P_{a,u}$and $P_{a,nu}$ denote the proportions of individuals of age with and without underlying conditions in China, respectively ^3^.

Analogously, the age-specific fatality ratios among laboratory-confirmed symptomatic cases for individuals with ($\mu_{a,u}$) and without ($\mu_{a,nu}$) underlying conditions are computed respectively as:

$\mu_{a,u} =v\cdot m_{u}^{ITA}\cdot$ $M_{a}$

$\mu_{a,nu} =v\cdot m_{nu}^{ITA}\cdot$ $M_{a}$

where,

- $m_{u}^{ITA}$ and$m_{nu}^{ITA}$ denotes the proportion of cases with fatal outcomes among symptomatic cases with and without underlying conditions as estimated from Lombardy data (Table S2) ^25,30,31^.
- $M_{a}$ denotes the age-specific fatality ratio among laboratory-confirmed symptomatic cases as estimated for China independently from the presence of underlying conditions ^29^.
- the scale factor $v$ is determined in such a way to minimize the root mean square error between $M_{a}$ and $\tilde{M_{a}}=$ $P_{a,u}\cdot\mu_{a,u} +P_{a,nu}\cdot\text{ }\mu_{a,nu}$.

Estimates were reported in Table S3.

**Table S2. Proportion of laboratory-confirmed symptomatic cases requiring hospitalizations and having fatal outcomes among patients with or without underlying conditions***

|  | With underlying conditions | Without underlying conditions | Total |
| --- | --- | --- | --- |
| Laboratory-confirmed symptomatic cases | 44446 | 44092 | 88538 |
| Laboratory-confirmed symptomatic cases requiring hospitalizations | 29593 | 17800 | 47393 |
| Laboratory-confirmed symptomatic cases with fatal outcomes | 13683 | 3095 | 16778 |
| Proportion of hospitalization among laboratory-confirmed symptomatic cases (%) | $h_{u}^{ITA}=$66.6 | $h_{nu}^{ITA}=$40.4 | 53.5 |
| Proportion of laboratory-confirmed symptomatic cases with fatal outcomes (%) | $m_{u}^{ITA}=$30.8 | $m_{nu}^{ITA}=$7 | 19 |

^*^ The data were obtained from the line list of COVID-19 patients in the Lombardy region of Italy, with underlying diseases including chronic respiratory disease, cardiovascular disease, metabolic disease and cancer ^25,30,31^.

**Table S3. Estimated hospitalization and death rates for individuals with and without underlying conditions in China.**

|  | With/without underlying conditions | With underlying conditions | Without underlying conditions |
| --- | --- | --- | --- |
| Proportion of laboratory-confirmed symptomatic cases requiring hospitalizations (%) | $\Delta_{a}$^29^ | $\sigma_{a,u}$ | $\sigma_{a,nu}$ |
| 0-19 years | 40 | 51.9 | 31.5 |
| 20-39 years | 29.2 | 37.9 | 23.0 |
| 40-59 years | 33.3 | 43.2 | 26.2 |
| 60+ years | 33.8 | 43.8 | 26.6 |
| Fatality ratio among laboratory-confirmed symptomatic cases (%) | $M_{a}$^29^ | $\mu_{a,u}$ | $\mu_{a,nu}$ |
| 0-19 years | 0.51 | 0.66 | 0.15 |
| 20-39 years | 0.65 | 0.84 | 0.19 |
| 40-59 years | 2.38 | 3.06 | 0.70 |
| 60+ years | 10.52 | 13.53 | 3.07 |

# Supplementary file 5. Data analysis

For each scenario, 200 stochastic model realizations were performed. The outcome of these simulations determined the distributions of the number of symptomatic infections, hospitalizations, ICU admissions, and deaths. 95% confidence intervals were defined as quantiles 0.025 and 0.975 of the estimated distributions. We used a Bayesian approach to estimate R_t_ from the time series of symptomatic cases by date of symptom onset and the distribution of the serial interval. The methods have been described previously ^11^.

To estimate R_t_, we assumed that the daily number of new cases (by date of symptom onset), including locally acquired infections L(t), can be approximated by a Poisson distribution according to the equation.

$$L(t)\sim Pois\left( R(t)\sum_{s=1}^{t} \varphi\left( s \right)C(t-s) \right)$$

Where,

- $C(t)$, with t from 0 to T, is the daily number of locally acquired new cases, by date of symptom onset;
- $R(t)$ is the net reproduction number at time t;
- $\varphi(s)$ is the distribution of the generation time (corresponding to the distribution of the serial interval) calculated at time s.

The likelihood ℒ of the observed time series of cases from day 1 to T conditional on $C(0)$ is thus given by

$$\mathcal{L=}\prod_{t=1}^{T} P\left( L\left( t \right);R(t)\sum_{s=1}^{t} \varphi\left( s \right)C(t-s) \right)$$

where $P(k; \lambda)$ is the probability mass function of a Poisson distribution (i.e., the probability of observing k events if these events occur with rate λ).

We used Metropolis-Hastings MCMC sampling to estimate the posterior distribution of $R(t)$. The Markov chains were run for 1,000,000 iterations, assuming non-informative prior distributions of $R(t)$ (flat distribution in the range (0-1000]). Convergence was checked by visual inspection by running multiple chains starting from different starting points. It should be noted that, when estimating $R(t)$, we excluded data at the start and end of the epidemic curve for these days with the first 5% quantile of daily number of symptomatic cases to deal with possible instability of $R(t)$ estimates due to small numbers of symptomatic cases. While for Rt=1.1 at the beginning of transmission, we do not exclude aforementioned data since the daily number of symptomatic cases remains tiny during the time frame of this study.

# References

1 Zhang, J. *et al.* Patterns of human social contact and contact with animals in Shanghai, China. *Sci. Rep.* **9**, 15141, doi:10.1038/s41598-019-51609-8 (2019).

2 United Nations, Department of Economic and Social Affairs, Population Division (2019). World Population Prospects 2019, Online Edition. Rev. 1.

3 Yang, J. *et al.* Who should be Prioritized for COVID-19 Vaccination in China? A Descriptive Study. *BMC Med,accepted*, doi:10.20944/preprints202009.0446.v1 (2020).

4 Hu, S. *et al.* Infectivity, susceptibility, and risk factors associated with SARS-CoV-2 transmission under intensive contact tracing in Hunan, China. *medRxiv*, 2020.2007.2023.20160317 (2020). <<http://medrxiv.org/content/early/2020/11/03/2020.07.23.20160317.abstract>>.

5 Sun, K. *et al.* Transmission heterogeneities, kinetics, and controllability of SARS-CoV-2. *Science*, eabe2424, doi:10.1126/science.abe2424.

6 Pan, A. *et al.* Association of Public Health Interventions With the Epidemiology of the COVID-19 Outbreak in Wuhan, China. *JAMA* **323**, 1915-1923, doi:10.1001/jama.2020.6130 (2020).

7 Li, Z. *et al.* Active case finding with case management: the key to tackling the COVID-19 pandemic. *Lancet* **396**, 63-70, doi:10.1016/s0140-6736(20)31278-2 (2020).

8 Xing, Y., Wong, G. W. K., Ni, W., Hu, X. & Xing, Q. Rapid Response to an Outbreak in Qingdao, China. *N. Engl. J. Med.* **383**, e129, doi:10.1056/NEJMc2032361 (2020).

9 Xinhua net,The COVID-19 outbreak in Beijing(2020); <http://www.xinhuanet.com/politics/2020-06/19/c_1126135352.htm>.

10 Li, Q. *et al.* Early Transmission Dynamics in Wuhan, China, of Novel Coronavirus–Infected Pneumonia. *N. Engl. J. Med.* **382**, 1199-1207, doi:10.1056/NEJMoa2001316 (2020).

11 Zhang, J. *et al.* Evolving epidemiology and transmission dynamics of coronavirus disease 2019 outside Hubei province, China: a descriptive and modelling study. *Lancet Infect. Dis.* **7**, 793-802, doi:10.1016/s1473-3099(20)30230-9 (2020).

12 The Central People's Government of the People's Republic of China, COVID-19 vaccine development, priority populations and pricing ... the lastest release(2020); <http://www.gov.cn/fuwu/2020-10/20/content_5552857.htm>.

13 The Central People's Government of the People's Republic of China,Report of H1N1 pandemic influenza vaccination from Ministry of Health(2020); <http://www.gov.cn/gzdt>.

14 Yang, J. *et al.* Who should be prioritized for COVID-19 vaccination in China? A descriptive study. *Preprints* (2020). <<https://www.preprints.org/manuscript/202009.0446/v1>>.

15 Xiao, A. *et al.* Dynamic Profile of RT-PCR Findings from 301 COVID-19 Patients in Wuhan, China: A Descriptive Study. *J Clin Virol* **127**, 104346, doi:10.1016/j.jcv.2020.104346 (2020).

16 S. Hu, W. Wang, Y. Wang, M. Litvinova, K. Luo, L. Ren, Q. Sun, X. Chen, G. Zeng, J. Li, L. Liang, Z. Deng, W. Zheng, M. Li, H. Yang, J. Guo, K. Wang, X. Chen, Z. Liu, H. Yan, H. Shi, Z. Chen, Y. Zhou, K. Sun, A. Vespignani, C. Viboud, L. Gao, M. Ajelli, H. Yu, Infectivity, susceptibility, and risk factors associated with SARS-CoV-2 transmission under intensive contact tracing in Hunan, China. medRxiv (2020), doi:10.1101/2020.07.23.20160317.

17 Xia, S. *et al.* Safety and immunogenicity of an inactivated SARS-CoV-2 vaccine, BBIBP-CorV: a randomised, double-blind, placebo-controlled, phase 1/2 trial. *Lancet Infect. Dis.* **21**, 39-51, doi:10.1016/S1473-3099(20)30831-8 (2021).

18 Sinopharm,Sinopharm COVID-19 vaccine licensed in China(2021); <http://www.sinopharm.com/s/1223-3763-38840.html>.

19 Callaway, E. COVID vaccine excitement builds as Moderna reports third positive result. *Nature* **587**, 337-338, doi: 10.1038/d41586-020-03248-7. (2020).

20 Voysey, M. *et al.* Safety and efficacy of the ChAdOx1 nCoV-19 vaccine (AZD1222) against SARS-CoV-2: an interim analysis of four randomised controlled trials in Brazil, South Africa, and the UK. *Lancet* **397**, 99-111, doi:10.1016/s0140-6736(20)32661-1 (2021).

21 Polack, F. P. *et al.* Safety and Efficacy of the BNT162b2 mRNA Covid-19 Vaccine. *N. Engl. J. Med.* **383**, 2603-2615, doi:10.1056/NEJMoa2034577 (2020).

22 Xia et al. "Safety and immunogenicity of an inactivated SARS-CoV-2 vaccine, BBIBP-CorV: a randomised, double-blind, placebo-controlled, phase 1/2 trial." The Lancet Infectious Diseases (2020).

23 Yang, P. *et al.* Influenza vaccine effectiveness against medically-attended influenza illness during the 2012–2013 season in Beijing, China. *Vaccine* **32**, 5285-5289, doi:10.1016/j.vaccine.2014.07.083 (2014).

24 Diekmann O, Heesterbeek JA, Metz JA. On the definition and the computation of the basic reproduction ratio R 0 in models for infectious diseases in heterogeneous populations. Journal of Mathematical Biology. 1990;28(4):365-82.

25 Poletti, P. *et al.* Probability of symptoms and critical disease after SARS-CoV-2 infection. *arXiv* (2020). <<https://arxiv.org/abs/2006.08471>>.

26 Deng, X. *et al.* Case fatality risk of the first pandemic wave of novel coronavirus disease 2019 (COVID-19) in China. *Clin Infect Dis*, ciaa578, doi: 10.1093/cid/ciaa578 (2020).

27 Guan, W. *et al.* Clinical Characteristics of Coronavirus Disease 2019 in China. *N. Engl. J. Med.* **382**, 1708-1720, doi:10.1056/NEJMoa2002032 (2020).

28 Xie, J. *et al.* Clinical characteristics and outcomes of critically ill patients with novel coronavirus infectious disease (COVID-19) in China: a retrospective multicenter study. *Intens Care Med* **46**, 1863–1872, doi:<https://doi.org/10.1007/s00134-020-06211-2> (2020).

29 Yang, J. *et al.* Disease burden and clinical severity of the first pandemic wave of COVID-19 in Wuhan, China. *Nat. Commun.* **11**, 5411, doi:10.1038/s41467-020-19238-2 (2020).

30 Poletti, P. *et al.* Age-specific SARS-CoV-2 infection fatality ratio and associated risk factors, Italy, February to April 2020. *Euro Surveill* **25**, 2001383, doi:10.2807/1560-7917.ES.2020.25.31.2001383 (2020).

31 Trentini, F. *et al.* Healthcare strain and intensive care during the COVID-19 outbreak in the Lombardy region: a retrospective observational study on 43,538 hospitalized patients. *medRxiv*, 2020.2011.2006.20149690 (2020). <<http://medrxiv.org/content/early/2020/11/07/2020.11.06.20149690.abstract>>.
